# Nanoparticle size distribution from inversion of wide angle X-ray total scattering data

**DOI:** 10.1038/s41598-020-69371-7

**Published:** 2020-07-29

**Authors:** Fabio Ferri, Federica Bertolotti, Antonietta Guagliardi, Norberto Masciocchi

**Affiliations:** 1 Department of Science and High Technology and To.Sca.Lab, University of Insubria, via Valleggio 11, 22100 Como, CO Italy; 2 Institute of Crystallography and To.Sca.Lab, Consiglio Nazionale Delle Ricerche, via Valleggio 11, 22100 Como, CO Italy

**Keywords:** Materials science, Nanoscience and technology

## Abstract

An increasingly important issue in nanoscience and nanotechnology is the accurate determination of nanoparticle sizing. Wide angle X-ray total scattering (WAXTS) data are frequently used to retrieve the Particle Size Distributions (PSDs) of nanocrystals of highly technological relevance; however, the PSD shape typically relies on an a-priori assumption. Here, we propose a modified version of the classical iterative Lucy-Richardson (LR) algorithm, which is simple, fast and highly reliable against noise, and demonstrate that the inversion of WAXTS data can be profitably used for recovering accurate PSD regardless of its shape. Computer simulations based on the use of the Debye Scattering Equation (DSE) modelling WAXTS data show that the algorithm is capable of recovering accurate PSDs even when the sample is made of a mixture of different polymorphs and/or exhibits microstrain effects. When applied to the inversion of WAXTS data taken on real samples, the method requires accurate modelling of the nanoparticle crystal structure, which includes structural defects, microstrain and surface induced distortions. Provided that this information is correctly fed to the program, the inversion algorithm reconstructs the WAXTS data with high accuracy and recovers highly robust (against noise) PSDs. Two examples reporting the characterization of Magnetite-Maghemite and commercial P25-Titania nanopowders, are discussed. We demonstrate that pre-assumption of wrong PSD shape leads to inaccurate number-based average sizes in highly polydisperse samples.

## Introduction

Nanoparticle sizing and nanoparticle structural characterization have become, over the last decades, topics of increasing interest due to their intimate relationship to nanoscience and nanotechnology^1–6^. Nanoparticles or NanoCrystals (NCs) usually exhibit a crystalline or a partially ordered atomic arrangement, with local structural distortions, defects, and remarkable surface effects (due to their large surface area to volume ratio) that can be tailored by proper functionalization. All these structural features, together with the NCs morphology and size, determine their physical–chemical properties and, ultimately, their functionality^7,8^. In particular, the detailed knowledge of the Particle Size Distribution (PSD) and Particle Size and Shape Distribution (PSSD) becomes an issue of extreme relevance when developing materials with advanced functionalities. This is for example the case of electroluminescent perovskites^9^, heterogeneous catalysts^10^, nanomaterials for optoelectronics^11^, photovoltaics^12^, drug delivery^13^, or in industrially relevant processes, such as filtration^14^, coating^15^, dyes and inks^16^, cosmetics^17^ and active pharmaceutical ingredients formulation^18^.

Among the experimental techniques that deal with nanoparticle sizing, imaging and scattering optical methods are among the most popular ones. Imaging methods, such as confocal optical^19^ or transmission electron^20^ microscopy, work by analyzing individually each single particle and therefore provide a direct measurement of the PSD and PSSD, but suffer from very poor statistics^21^ (unless the measurements are repeated many times). Conversely, when the measurements are carried out in the reciprocal space as it occurs with scattering methods, such as Static Light Scattering (SLS), Dynamic Light Scattering (DLS), Small Angle X-ray Scattering (SAXS) and Wide Angle X-ray Scattering (WAXS), the statistics is very high because a very large number of particles are examined at the same time. Depending on the length scales being probed, these techniques provide different (and complementary) information on the nanoparticles. For example, SLS, DLS and SAXS give useful information on the PSD and PSSD in the micro- to nano-range sizes, but they are totally blind to the nanoparticle structure such as atomic arrangement and crystal defectiveness. The latter ones can be investigated by using WAXS techniques and in particular the classical method called X-ray Powder Diffraction (XRPD) that works by analyzing Bragg peak positions, widths and integrated intensities. XRPD typically gives information on composition, average crystal structure and (via Scherrer’s equation) average crystal size of the (defect-free) nanoparticles, but no information about PSD and PSSD is normally provided. All the methods (including X-ray absorption, photoluminescence, NMR, mass spectrometry, and others) used for the characterization of NCs in terms of composition, structure, PSD and PSSD are presented in Ref.^22,23^.

Recently, with the advent of the methods called Wide Angle X-ray Total Scattering (WAXTS), the possibility of simultaneously characterizing the nanoparticle structure and PSSD has become at hand within a single technique^24,25^. The methods work by measuring the total X-ray pattern scattered by the sample at wide angles and, by exploiting information coming not only from peak positions and integrated intensities (as done in standard XRPD) but also from diffuse scattering and sample-dependent peak width and shape, particle composition, structure, defects, morphology and size distribution can be recovered^26–31^.

The analysis of WAXTS data can be carried out either in the direct space by Fourier transforming the Intensity profile and recovering the Pair Distribution Function (PDF) of the sample^32,33^, or in the reciprocal space by directly analyzing the experimental scattering data, which are described by the Debye-Scattering Equation (DSE)^24,34^. In both cases, the analysis relies on atomic scale models of NCs and, generally, on the pre-assumption of a (discrete) analytical function describing the sample PSD. In this article, we will focus on the DSE method (named as WAXTS-DSE) and demonstrate that PSD can be recovered from experimental data without any a priori assumption by using a modified version of the well-known Lucy^35^-Richardson^36^ algorithm, which has never been used before for this kind of data analysis.

### Theoretical background

The Debye Scattering Equation (DSE)^34^ provides the intensity scattered by randomly oriented monodisperse (i.e. equal size) nanoparticles composed by N atoms whose interatomic distances between atomic pairs are known:

1 $$I\left(Q\right)=\sum_{i}^{N}{f}_{i}^{2}\left(Q\right){o}_{i}^{2} +2\sum_{i,j>i}^{N}{f}_{j}(Q){f}_{i}(Q){T}_{j}(Q){T}_{i}(Q){o}_{j}{o}_{i} \frac{\sin(Q{d}_{ij})}{Q{d}_{ij}}$$

where $Q=(4\pi /\lambda) \sin(\theta )$ is the magnitude of the scattering vector, *θ* is half of the scattering angle, λ is the radiation wavelength, ${f}_{i}$ is the X-ray atomic form factor of element i, ${d}_{ij}$ is the interatomic distance between atoms *i* and *j*, *T* and *o* are the (isotropic) Debye–Waller thermal displacement parameter and the site occupancy factor associated to each atomic species, respectively. The first term of Eq. (1) is given by the sum of the intensities scattered from all the atoms composing the nanoparticle, whereas the second term accounts for the interference between the waves scattered by all the atoms within the NP.

When the sample is polydisperse and/or polyphasic, Eq. (1) must be summed over nanoparticles of different sizes and/or phases. This task can be overwhelming in terms of computational times because the number of terms appearing in the interference sums of Eq. (1) scales as the square of the number of atoms, which, in turn, grows very rapidly with particle size ($\sim {d}^{3}$ for spherical particles). A way out to cope with this problem is using a suitable algorithm that, relying on a highly reduced number (by orders of magnitudes^37^) of interatomic distances, can compute Eq. (1) with acceptable computational times, without lacking any accuracy^38^. Such algorithm is implemented in the recently published DEBUSSY suite of programs^39^. Practically, the WAXTS-DSE study is performed by creating, in a preliminary and independent step, a database of Gaussian sampled interatomic distances for each phase from which the scattering profiles of a set of nanoparticles of different sizes and phases are calculated. Specific tests^38^ have demonstrated that the agreement between DSE simulations calculated using Gaussian sampled and true interatomic distances, results in relative errors at the 10^–6^ level, (or even smaller, depending on the sampling step, however at expenses of the computational time) well below the noise level of the experimental data. Based on this consideration, we can safely state that using sampled distances does not affect the accuracy of the inversion algorithm later discussed. In the following, we will always use the sampled distances whenever implementing Eq. (1).

Once the scattering profile of each nanoparticle is available, the WAXTS-DSE method works by fitting the data on the assumption that the (number) PSD of each phase is described by a LogNormal distribution, in which the first two momenta (average and variance) and the relative weight fraction are retrieved by standard χ^2^ minimization. Therefore, the WAXTS-DSE method pivots on a strong assumption, namely the shape of the PSD, which if not appropriate, may affect significantly the results. Two examples of the errors introduced by this assumption when the distribution shape is fairly different from that of a LogNormal, are reported and discussed in the Supplementary Information 1 (SI) file, section 6.

The request of a pre-assumed PDS shape could be removed if, instead of fitting, one would invert the data. In this article, we propose the inversion of WAXTS-DSE data that characterizes a sample made of collection(s) of nanocrystals of different sizes and shapes, for a single material or a mixture of phases. Although the different common morphologies might be described by size distributions that require more than one size parameter (for example, length and diameter for cylinders or three sides for prismatic platelets), in this work we consider only monovariate distributions as a proof of concept. For a monovariate distribution, the nanoparticle size is pin-pointed by a single size parameter, namely the diameter of the equivalent sphere with a volume equal to the particle volume. Thus, when the sample is composed by $P$ different phases and each phase is made of ${M}_{p}$ nanoparticles of different diameters, the intensity scattered at a given wavevector ${Q}_{i}$ can be written as

2 $$I\left({Q}_{i}\right)=\sum_{p=1}^{P} \sum_{j=1}^{{M}_{p}}{ N}_{p}\left({d}_{p,j}\right) {I}_{p}\left({Q}_{i},{d}_{p,j}\right) +\alpha {I}_{0}\left({Q}_{i}\right)$$

where ${N}_{p}\left({d}_{p,j}\right)$ is the Number diameter distribution of the p-th phase and ${I}_{p}\left({Q}_{i},{d}_{p,j}\right)$ is the intensity scattered at ${Q}_{i}$ by a nanoparticle of size ${d}_{p,j}$ belonging to the p-th phase. In Eq. (2) we have also added the term $\alpha {I}_{0}\left({Q}_{i}\right)$ which is a background contribution associated either to an amorphous component present in the sample or, as in the case of colloidal nanoparticles, is due to the solvent. Note that, since the shape of ${I}_{0}\left({Q}_{i}\right)$ is supposed to be known whereas the weight factor α is not, the last term of Eq. (2) can be formally interpreted as the contribution of an equivalent extra phase made of single sized particles.

When the measurements are taken at $N$ different wavevectors ${Q}_{i}$ ($i=1,2\dots N$), Eq. (2) represents a set of N algebraic linear equations in which $I\left({Q}_{i}\right)$ are the known terms provided by the experiment and $\left\{{N}_{p}\left({d}_{p,j}\right)\right\} \;{\text{and}}\; \alpha$ are the $M={\sum }_{p=1}^{P}{M}_{p}+1$ unknowns. Thus, Eq. (2) can be compactly rewritten as

3 $$I\left({Q}_{i}\right)= \sum_{k=1}^{M}{A}_{i,k}{ N}_{k}$$

where ${N}_{k}$ ($k=1, 2,\dots ,M$) are the overall concatenated unknowns (all sizes of all phases) plus the amplitude α of the background term, whereas ${A}_{i,k}$ is a $N\times M$ matrix built by merging together, column by column, the matrices ${I}_{p}\left({Q}_{i},{d}_{p,j}\right)$ and the profile ${I}_{0}\left({Q}_{i}\right)$. Equation (3) is a typical example of an ill-conditioned problem, meaning that, in the presence of even a very small (but unavoidable) level of noise on $I\left({Q}_{i}\right)$, quite different distributions can reconstruct the data to the same level of statistical accuracy. As a consequence, the solution of Eq. (3) is not a trivial task and a suitable inversion algorithm has to be adopted. The algorithm used in this work is described in the next paragraph.

### Inversion algorithm

The inversion of WAXTS-DSE data is herein carried out by using a modified version of the Lucy-Richardson (LR) algorithm, which was proposed long time ago by Lucy^35^ and Richardson^36^ in the field of image restoration. The LR method is based on a simple iterative nonlinear algorithm that ensures non-negativity of the solutions, is rather robust (but not immune) against noise and, provided that the iterative procedure is stopped after a properly chosen number of steps, does not require any parameter to be optimized^40^. The LR algorithm appears to be quite suitable for dealing with the inversion of WAXTS-DSE data because: (i) the data to be inverted are usually taken at high *Q*-resolution, over a large *Q*-range^40^; (ii) the noise on the data is expected to be described by a Poisson statistics^41^, with a high signal-to-noise ratio (SNR); (iii), the kernels ${I}_{p}\left({Q}_{i},{d}_{p,j}\right)$ associated to Eq. (1) are highly structured^40^ with the presence of a large number of relatively narrow and differently shaped peaks. These three features make the inversion of Eq. (3) not a severely ill-posed problem because, as long as the SNR on the data is high enough that the Intensity profiles of two adjacent size classes are significantly different, the inversion algorithm can work properly without introducing artefacts. A detailed analysis on the ill-posedness of the WAXTS-DSE data inversion problem is reported in Supplementary Information 1, section 11.

The implementation of the modified LR algorithm to the inversion of the WAXTS-DSE data described by Eq. (3) works as follows. Let us suppose that ${N}_{k}^{r}$ is the concatenated size distribution recovered after *r* iterations. Thus, at the $\left(r+1\right)$ step the distribution is corrected as

4 $$\begin{aligned}{N}_{k}^{r+1}&={\Lambda }_{{\omega }_{p}}\left\{{N}_{k}^{r}\sum_{i=1}^{N}{W}_{i,k}\frac{{I}^{m}({Q}_{i})}{{I}^{r}({Q}_{i})}\right\} \\ { W}_{i,k} &=\frac{{A}_{i,k}}{\sum_{i=1}^{N}{A}_{i,k}}\end{aligned}$$

where ${I}^{m}({Q}_{i})$ is the measured scattering profile, ${I}^{r}({Q}_{i})$ is the profile reconstructed after *r* iterations by inserting ${N}_{k}^{r}$ into Eq. (3) and ${\Lambda }_{{\omega }_{p}}$ are phase-dependent 3-points triangular operators that perform a smoothing of ${N}_{k}^{r}$ before passing to the next iteration. The amplitude of the lateral points in the ${\Lambda }_{{\omega }_{p}}$ operators are set by the parameters ${\omega }_{p}$, whose typical values are between ${\sim 10}^{-1}{-}{10}^{-7}$, depending on distribution width and SNR (see Supplementary Information 1, section 1). The introduction of the smoothing procedure is the novelty of our algorithm with respect to the original LR algorithm; the latter one suffers from some instabilities against noise^42^, which produce recovered distributions with an unphysical spiky appearance (See Supplementary Information 1, section 1). The iterative smoothing procedure acts as a regularization scheme^42^ capable of removing such a spurious and unphysical feature. Details on the optimization of the smoothing parameters ${\omega }_{p}$ and a comparison with the original LR algorithm are discussed in Supplementary Information 1, section 1.

Notice that in Eq. (4) the population of each class is corrected (except for the smoothing operation) independently of the populations of all the other classes and the correction is based only on the (weighted) ratios ${I}^{m}({Q}_{i})/{I}^{r}({Q}_{i})$ between the measured and reconstructed data. These two features make the algorithm very simple and fast. The iterative procedure is initiated by starting from a flat distribution (${N}_{k}^{0}=1$ for any $k$) and is stopped at $r={r}_{*}$ when the goodness of fit (GOF) parameter defined as

5 $$\text{GOF}\left(r\right)=\sqrt{\frac{1}{N} \sum_{i=1}^{N}{\left(\frac{{I}^{m}\left({Q}_{i}\right)-{I}^{r}\left({Q}_{i}\right)}{{\sigma }_{i}}\right)}^{2}}$$

where ${\sigma }_{i}$ are the experimental error bars, attains a minimum or becomes stationary. Details on the stopping criteria are reported in Supplementary Information 1, section 2.

**Figure 1:**
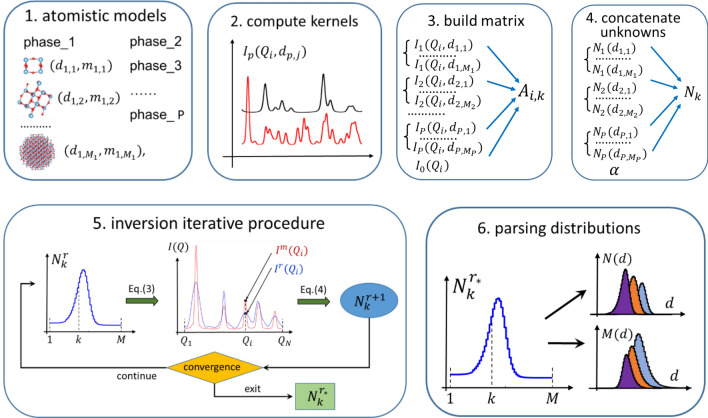
Sketch of data analysis carried out in 6 steps. (1) Build the atomistic model of each phase; (2) compute the scattering profile of all the nanocrystals of each phase ${I}_{p}\left({Q}_{i},{d}_{p,j}\right)$ by using the DEBUSSY suite; (3) build the inversion matrix ${A}_{i,k}$; (4) concatenate all the unknowns ${N}_{p}\left({d}_{p,j}\right)$ into a single array ${N}_{k}$; (5) run the iterative inversion procedure and correct ${N}_{k}^{r}$ by comparing the measured data ${I}^{m}\left({Q}_{i}\right)$ with the reconstructed ones ${I}^{r}\left({Q}_{i}\right)$. At convergence, the final solution is ${N}_{k}^{{r}_{*}}$; (6) recover the final number and mass diameter distributions by parsing the solution ${N}_{k}^{{r}_{*}}$. Mass distributions are computed from number distributions as ${M}_{p}\left({d}_{p,j}\right)={ N}_{p}\left({d}_{p,j}\right) {m}_{p,j}.$

Figure 1 provides a schematic summary of all the steps followed in a typical data analysis. First, the atomistic models of the various crystal phases are built, resulting in the encoding of all the interatomic distances, diameters ${d}_{p,j}$ and masses ${m}_{p,j}$ of each nanocrystal (step 1); then, by using the DEBUSSY suite, the intensity profiles ${I}_{p}\left({Q}_{i},{d}_{p,j}\right)$ of all the nanocrystals are computed (step 2) and the inversion matrix ${A}_{i,k}$ is assembled by merging together (column by column) all the matrices ${I}_{p}\left({Q}_{i},{d}_{p,j}\right)$ plus the background profile ${I}_{0}\left({Q}_{i}\right)$ (step 3); similarly, all the unknowns ${N}_{p}\left({d}_{p,j}\right)$ plus the amplitude $\alpha$ of the background profile are concatenated together in a single 1D-unknown array ${N}_{k}$ (step 4); at this point (step 5) the iterative inversion procedure is run and ${N}_{k}^{r}$ is corrected by comparing the measured data ${I}^{m}\left({Q}_{i}\right) ($red line) with the reconstructed ones ${I}^{r}({Q}_{i})$ (blue line). When convergence is attained, the final (concatenated) solution ${N}_{k}^{{r}_{*}}$ is found; finally (step 6), the number diameter distributions of each phase ${N}_{p}\left({d}_{p,j}\right)$ are recovered by parsing the solution ${N}_{k}^{{r}_{*}}$. Mass distributions are computed as ${M}_{p}\left({d}_{p,j}\right)={ N}_{p}\left({d}_{p,j}\right) {m}_{p,j}.$

### Numerical simulations

The proper functioning, efficiency and accuracy of the inversion algorithm applied to WAXTS-DSE were tested by using *in-silico* simulations. For each simulation, we built the atomistic models of various nanocrystal phases, generated a plausible background profile and computed the matrix ${A}_{i,k}$. Then, according to a set of pre-defined “input” distributions ${N}_{p}\left({d}_{p,j}\right)$ and corresponding concatenated input array ${N}_{k}^{inp}$, the ideal (noiseless) scattering profile $I\left({Q}_{i}\right)$ was computed by using Eq. (3). Such data were passed through a Poisson filter so to produce realistic noisy “input” data to be inverted by means of Eq. (4). The inversion was carried out by using the same kernel functions used for generating the noiseless scattering profile $I\left({Q}_{i}\right).$ At the end of the inversion procedure, the final recovered data and distributions are compared with the input ones.

#### Mixture of polymorphic TiO_2_ NCs

**Figure 2:**
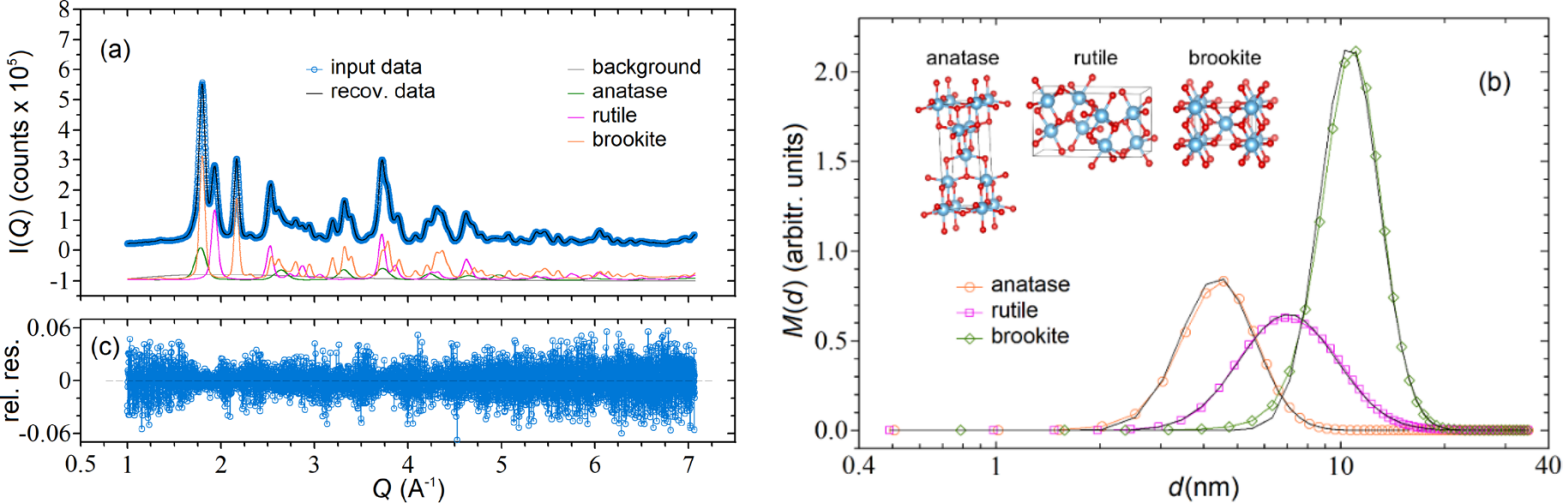
(**a**) Simulated input (blue circles) and reconstructed (black curve) WAXTS data for a TiO_2_ sample composed by the three phases: anatase, rutile and brookite. For the sake of clarity, the single phases (noiseless) contributions and a background profile have been shifted by − 1. The noise added to the data was generated according to a Poissonian distribution; (**b**) input (black curves) and recovered (colour curves) mass distributions of the three phases obtained by inverting the input data of (**a**); (**c**) relative residuals [(*data-fit*)/*fit*] between recovered and input data. The peak-to-peak fluctuations of ($\sim \pm 0.06$) are consistent with the noise level ($SNR\sim 300$) used in the simulation.

Figure 2 shows an example of this kind of simulations in which a TiO_2_ sample is composed by a mixture of nanocrystals of the three common polymorphs *anatase*, *rutile*, and *brookite*^43^. Each phase is supposed to be characterized by a LogNormal (number) distribution of monovariate spheroidal nanoparticles of average size  ⟨ *d* ⟩ _n_, standard deviation σ_n_ and relative (number) concentration c_n_ (see Supplementary Information 1, section 3 for all parameter values). Figure 2a shows the noisy input scattering profile ${I}^{m}\left(Q\right)$ associated to such a sample (blue circles) obtained by summing the noiseless profiles of each single phase [anatase (green), rutile (magenta), brookite (orange) curves] to the background profile (grey curve) and adding a Poisson distributed noise. Note that the scattering profiles of the three phases exhibit quite different peak shapes and positions, a feature that makes the inversion problem clearly not ill-posed (see Supplementary Information 1, section 11) and renders the inversion algorithm very efficient in recovering the PSD of each single phase. The sample concentration was chosen so to have a maximum count of $\sim {10}^{5}{-}{10}^{6}$ (equal to the typical count encountered at synchrotron facilities), which corresponds to an average $SNR={\left({\sum }_{i=1}^{N}{I}_{i}^{2}/{\sum }_{i=1}^{N}{\sigma }_{i}^{2}\right)}^{1/2}\sim 300$. The black curve passing through the data is the reconstructed profile according to the recovered mass distributions shown in Fig. 2b. The latter ones were obtained by setting ${\omega }_{p}={10}^{-4}$ for all the phases and stopping the iterative procedure at the minimum GOF $\sim 1.01$ after $\sim 1.8\times {10}^{4}$ iterations, where the matching between the input and reconstructed data is quite good, with non-systematic relative residuals [(*data-fit*)/*fit*] (Fig. 2c).

The recovered mass distributions are shown in Fig. 2b. The matching between recovered and input PSDs is excellent, as also witnessed by the fairly good agreement between the input and recovered mass based parameters reported in Table 1 (comparison between number distribution parameters are reported in Supplementary Information 1, section 3). Parallel tests considering the same combination of phases plus background and higher noise levels systematically recover the distributions shown in Fig. 2b, demonstrating the robustness of the inversion algorithm (see Supplementary Information 1, section 5).

**Table 1:** Comparison between input and recovered parameters for the *mass* distributions of Fig. 2.

TiO_2_ phase	Input			Recovered		
	⟨ *d* ⟩ _m_ (nm)	σ_m_ (nm)	c_m_ (%)	⟨ *d* ⟩_m_ (nm)	σ_m_ (nm)	c_m_ (%)
Anatase	4.80	1.20	0.173	4.81	1.22	0.173
Rutile	8.23	2.74	0.291	8.23	2.76	0.291
Brookite	11.25	2.25	0.536	11.25	2.31	0.537
Background (a.u.)	–	–	1	–	–	0.998

#### Highly strained Fe_5_Te_4_ NCs

The second test was aimed at ascertaining the capability of the inversion method to retrieve the correct PSD when the sample is not a mixture of different phases (that have different crystalline structures and therefore quite different peaks shapes and positions), but is characterized by microstrain distortions that affect only peaks widths and shapes, leaving unchanged their positions. In this case the inversion task is expected to be much more difficult and, depending on noise level, microstrain type and extent, and size distribution shape, the results may or may not be reliable. For this test we used a recently characterized nanocrystalline iron-rich telluride material, Fe_5_Te_4_, which has been found to exhibit structural distortions (microstrain) that derive from the mechano-chemical synthesis^44^. Microstrain along a given crystallographic direction $x$ can be quantified as the ratio between the root mean square modulation ${\langle {\Delta x}^{2}\rangle }^{1/2}$ of the interatomic distances and their average value $\langle x\rangle$, i.e. through the parameter ${\epsilon }_{x}={\langle {\Delta x}^{2}\rangle }^{1/2}/\langle x\rangle$. When ${\epsilon }_{x}$ is equal along all the three lattice vectors, a*, b, c,* the microstrain is isotropic, otherwise is anisotropic. Traditional methods for estimating isotropic and anisotropic strain parameters rely on single peak or full pattern analysis of the angular dependence of peak widths. Typical values for this and other materials are in the range ${\epsilon }_{x}\sim 0.1{-}1\%$ ^45^.

In this test, the input WAXTS-DSE data were generated according to a collection of anisotropically (${\epsilon }_{ab}=0.85\%, {\epsilon }_{c}=0.35\%$) microstrained Fe_5_Te_4_ nanoparticles characterized by a LogNormal distribution in diameters (${\langle d\rangle }_{n}=10\;\text{nm}$_,_ ${\sigma }_{n}/{\langle d\rangle }_{n}=20\%$). The inversion was carried out by allowing the program to use “three sets of phases”, namely the ones with no strain, the correct anisotropic strain (${\epsilon }_{ab}=0.85\%, {\epsilon }_{c}=0.35\%$) and an isotropic strain (${\epsilon }_{abc}=0.70\%$). As for the test on TiO_2_ NCs, we set ${\omega }_{p}=5\times {10}^{-5}$ for all the phases and stopped the iterative procedure at the minimum GOF ($\sim 1.002$ after $\sim 5\times {10}^{5}$ iterations).

**Figure 3:**
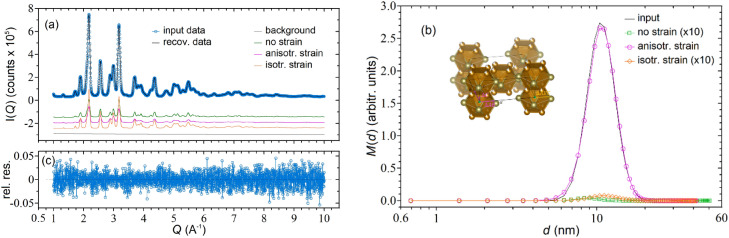
(**a**) Simulated input (blue circles) and reconstructed (black curve) WAXTS data for an anisotropic microstrained Fe_5_Te_4_ sample. The inversion was performed by letting the algorithm to sum the contributions of three phases (no strain, isotropic and anisotropic strain) plus a background profile. The noise added to the data was generated according to a Poissonian distribution; (**b**) Simulated (black curves) and mass distributions of the three phases recovered by inverting the WAXTS data of (**a**). For improving visibility, the no strain and isotropic strain curves have been amplified by a factor 10; (**c**) relative residuals between reconstructed and input data.

Figure 3a shows that the matching between the input (blue circles) and recovered (black line) data is excellent with non-systematic residuals (Fig. 3c), in spite of the fact that the scattering profile of the three used phases are quite similar, in term of both peak positions and shapes. The goodness of the inversion is also witnessed by the accuracy of the PDS reconstruction (Fig. 3b), in which only the (correct) single phase used for generating the data (anisotropic microstrain distribution) is sorted out, whereas the other two phases almost totally vanish. Quantitatively, these results are reported in Table 2, where one can appreciate the excellent matching between the (mass) input and recovered distribution parameters (rows 4–7)).

**Table 2:** Comparison between input and recovered parameters for the *mass* distributions of Fig. 3 (rows 4–7); recovered parameters when the inversion is carried out by using only one phase with the wrong strain (rows 8–9).

Fe_5_Te_4_ phase	Input			Recovered			
	⟨ *d* ⟩ _m_ (nm)	σ_m_ (nm)	c_m_ (%)	⟨ *d* ⟩_m_ (nm)	σ_m_ (nm)	c_m_ (%)	GOF
No-strain	–	–	0	9.13	1.62	0.001	1.002
Aniso-strain	11.25	2.25	1	11.26	2.26	0.996	
Iso-strain	–	–	0	11.53	2.27	0.003	
Background (a.u.)	–	–	1	–	–	0.998	
No-strain only	11.25	2.25	1	8.40	1.84	1	13.5
Iso-strain only	11.25	2.25	1	11.55	2.60	1	5.93

For this test, one may question how important is to define a reasonable set of strain parameters. Raw data provide information on these values, considering the Q-dependent and *hkl*-dependent broadening of the diffraction peaks, which has a different functional dependence than that attributable to finite size. When using the DSE approach, spanning the goodness-of-fit (hyper) surface of strain parameters is a viable option, as done by us in ref^44^. In any case, upon assuming wrong strain levels and inverting the data by using only the no-strain or the iso-strain phases, the accuracy of the signal reconstructions deteriorates significantly (large GOFs) and non-negligible errors are made for the parameters recovery (see rows 8–9 of Table 2). For the no-strain case, the remarkable underestimation of ${\langle d\rangle }_{m}$ is due to smaller sizes counterbalancing the lack of strain broadening. For the iso-strain case, the errors are somewhat reduced, but the recovered distribution is quite different from the input one with the presence of an extra broad peak at small sizes (data not shown).

### Inversion of experimental data

We tested our modified LR algorithm on experimental WAXTS data measured on real samples by using synchrotron X-rays^46^. The results of the inversion algorithm are discussed in comparison to those provided by the DSE-based analysis using the DEBUSSY suite. Experimental details are given in Supplementary Information 1, section 6.

When dealing with data collected on real samples, it is extremely important to model the nanocrystal structure with very high accuracy, taking into account any possible deviations from spatial periodicity due to finite-size and/or surface-driven structural distortions, microstrain, size-dependent lattice parameters, atomic thermal relaxation and any other kind of defects. Indeed, any discrepancy between the modelled and actual structure as well as any inaccuracy in the (shape of) background signal introduce systematic errors in the kernel functions ${I}_{p}\left({Q}_{i},{d}_{p,j}\right)$ that might produce not negligible artefacts in the recovered distribution. Similarly, it is crucial to take into account any (sample independent) bias introduced by the experimental setup, such as the presence of a blank signal due to the glass capillary scattering (which has to be removed prior to the analysis), or the peak broadening due to the finite instrumental response function, which might be particularly nasty for large sizes ($\ge 10{-}20\,\text{nm}$) and low-angular resolution instrumentation. Therefore, the usage of high-resolution experimental set-ups, such as dedicated synchrotron beamlines, outperforms laboratory sources, as it widens the nanoparticle size range which can be safely studied, up to tens of nanometers, i.e. to values which would be significantly underestimated if proper corrections of instrumental broadening are not performed. A few examples of the distribution artefacts arising in these situations are reported in Supplementary Information 1, section 5.

#### Magnetite-maghemite (Fe_3_O_4_–Fe_2_O_3_) NCs

**Figure 4:**
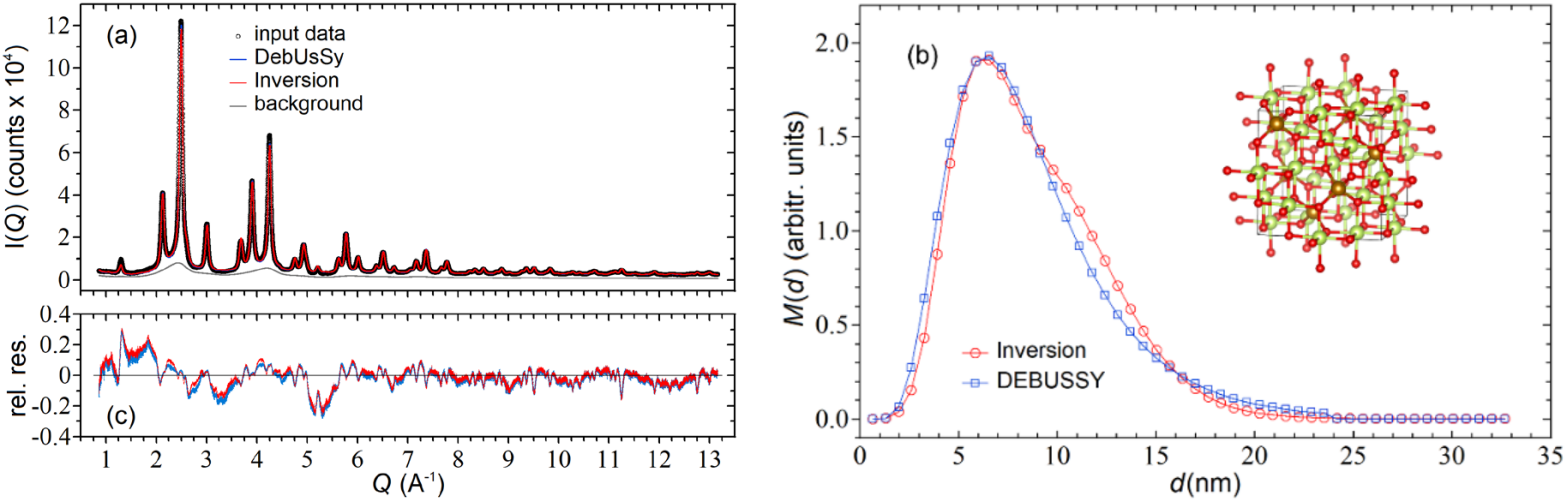
(**a**) Experimental (black circles) and reconstructed inversion (red line) WAXTS-DSE data for core–shell Magnetite-Maghemite (Fe_3_O_4_–γ-Fe_2_O_3_) nanocrystals measured with synchrotron X-ray radiation. The blue line indicates the DEBUSSY analysis. The grey line is the scattering profile of the amorphous phase used as a background in both analysis; (**b**) mass diameter distributions recovered with the inversion procedure and the DEBUSSY analysis. In the crystal lattice cell oxygen ions are in red, iron ions in tetrahedral sites in gold, and iron ions in octahedral coordination in light green (see also Supplementary Information 1, section 7); (**c**) relative residuals between the experimental and reconstructed data.

Herein we present the outcome of the inversion of synchrotron WAXTS data collected on a powder sample of superparamagnetic iron oxide nanoparticles exhibiting partial oxidation of Fe^2+^ ions, resulting into chemically inhomogeneous core–shell Magnetite-Maghemite (MM) nanocrystals. Worthy of note, the conditions of their preparations (co-precipitation) have been frequently reported, by a variety of experimental techniques, to follow a LogNormal size distribution law as in^47–50^. This is also valid for nanoparticle vapour phase growth method employed for fumed titania, discussed in the following sub-section (see for example Refs.^51,52^), thus, not unexpectedly, the conventional DEBUSSY approach used in both cases provides satisfactory results, but was further challenged by the present inversion method. The DEBUSSY analysis of such a sample [labelled A1 in Ref.^53^] relied on a monovariate (LogNormal) size distribution of spherically shaped NCs (cubic crystal structure, space group Fd-3m) where the size-dependent MM stoichiometry, the lattice parameters and Debye–Waller isotropic thermal factors were globally optimized against the experimental data; the contribution of an amorphous phase (probably two-line ferrihydrite) necessary for accurately fitting the data, was independently measured and used as a background signal. Overall, the DSE-based model allowed to fit the WAXTS data with very high accuracy (Fig. 4a) and to quantitatively correlate the structural to the magnetic properties.

In order to make the MM nanocrystals a test-model for the inversion algorithm (as much as possible) irrespective of the DEBUSSY-defined model, we generated a population of spherical NCs (up to a diameter of $\sim 33 \,\text{nm}$) using an average crystal structure model derived from previously optimized parameters (see Supplementary Information 1, section 7 for details). Worth of note, these values might be provided also by a standard Rietveld fit of the experimental pattern that does not rely on any size distribution information. We inverted the data by setting ${\omega }_{p}={2\times 10}^{-3}$ and stopping the iterative procedure at the minimum GOF $\sim 4.9$ after $\sim 5.0\times {10}^{3}$ iterations. The recovered mass size distribution is quite similar to the LogNormal recovered with the DEBUSSY analysis (Fig. 4b). Thus, the two methods provide consistent results, as also demonstrated by the remarkable similarity between the two reconstructed WAXTS data (Fig. 4a,c). A quantitative comparison between the mass distribution parameters recovered with the inversion procedure and DEBUSSY analysis is reported in Table 3.

**Table 3:** Comparison between DEBUSSY and Inversion results for the Magnetite-Maghemite data of Fig. 4.

	⟨ *d* ⟩_m_ (nm)	σ_m_ (nm)	Background (a.u.)	GOF
DEBUSSY	8.55	3.81	0.346	4.8
Inversion	8.70	3.50	0.291	4.9

#### Commercial titania (TiO_2_) NCs

The second test regards the characterization of a commercial “P25” Titania powder sample (Sigma-Aldrich, CAS # 13463-67-7, product # 71467) that, according to the product technical specifications, contains primary particles of $\sim 21\,\text{nm}$ (TEM) and is known to be a mixture of anatase (dominant) and rutile (minor) polymorphs.

For anatase we used the structural data available in literature further optimized by a Rietveld refinement using the Topas program (see Supplementary Information 1, section 8) and computed the kernel functions ${I}_{p}\left({Q}_{i},{d}_{p,j}\right)$ via the DEBUSSY suite with the exact DSE (Eq. 1) of spherical NCs up to diameters of $\sim 80$ nm.

**Figure 5:**
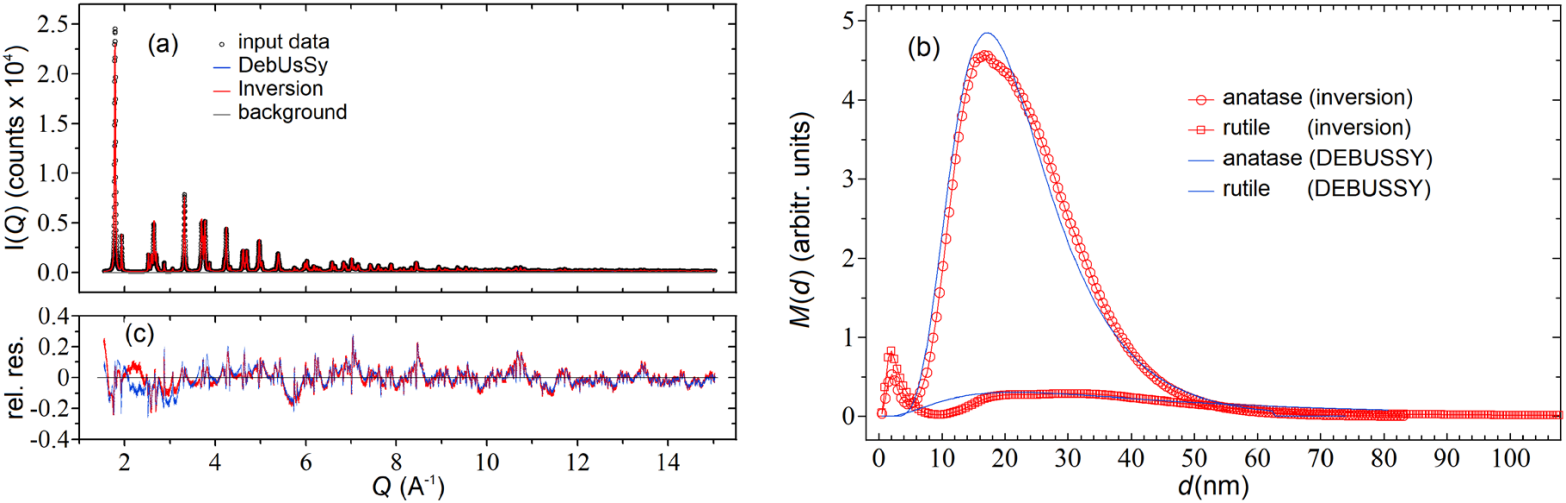
(**a**) Experimental (black circles) and reconstructed inversion (red line) synchrotron WAXTS-DSE data for a commercial Titania (TiO_2_) composed by nanopowder polymorphs of anatase and rutile. The blue line indicates the DEBUSSY analysis. The grey line is a constant background profile used for both analysis; (**b**) mass diameter distributions of the anatase and rutile phases recovered with the inversion procedure (red symbols) and the DEBUSSY analysis (blue curves); (**b**) relative residuals between the experimental and reconstructed data.

For the rutile phase, the occurrence of very large sizes ($\ge 100\,\text{nm}$) emerging from this analysis discouraged the use of the DSE (Eq. 1) for the computation of kernel functions, due to computational time issues. We resorted to an alternative approach based on the Rietveld method and Topas^54^ program (see Supplementary Information 1, section 8), which allowed us to compute the (approximate) functions ${I}_{p}\left({Q}_{i},{d}_{p,j}\right)$ of spherical NCs up to diameters of $\sim 200 \,\text{nm}$. The inversion was carried out by setting ${\omega }_{p}={2\times 10}^{-3}$ for both phases, using a constant background to improve the quality of data reconstruction, and stopping the iterative procedure at the minimum GOF $\sim 14.8$ after $\sim 1.7\times {10}^{3}$ iterations. The matching between the reconstructed and experimental WAXTS data is rather accurate (Fig. 5a) as also shown by the relative residuals plot reported in Fig. 5c. For comparison, we report in the two figures also the data and the residuals obtained with the DEBUSSY analysis, which appears to be quite similar to our method.

The distributions of the two phases recovered by the inversion procedure are shown in Fig. 5b (symbols) together with the distributions obtained from the DEBUSSY analysis (solid curves). Notice that the distributions recovered from our inversion algorithm presents two spurious peaks at very small sizes, which are artefacts arising probably from imperfect modelling of the rutile phase or from the use of a somewhat inaccurate background (See Supplementary Information 1, section 5). Nevertheless, the presence of these two peaks does not jeopardize the reliability of the recovered ${\langle d\rangle }_{m}$ and ${\sigma }_{m}$ parameters that, in the distributions with and without the peaks, differ, respectively, by $\sim 1\%$ and $\sim 2\%$ for anatase and by $\sim 20\%$ and $\sim 10\%$ for rutile. In conclusion, we can say that, also in this case, the inversion and the DEBUSSY analyses provide very similar results, as evidenced by the quantitative comparison reported in Table 4.

**Table 4:** Comparison between DEBUSSY and Inversion results for the Titania data of Fig. 5.

Phase	Inversion (GOF = 18.8)			DEBUSSY (GOF = 15.3)		
	< *d* > _m_ (nm)	σ_m_ (nm)	c_m_ (%)	< *d* > _m_ (nm)	σ_m_ (nm)	c_m_ (%)
Anatase	22.7	9.93	0.87	23.2	10.2	0.88
Rutile	36.7	25.5	0.13	34.6	19.3	0.12

## Conclusions

We have shown that WAXTS data taken on a variety of nanocrystal samples can be profitably processed by using a simple and fast inversion algorithm that allows to recover the PSDs of all the phases composing the sample. The algorithm used in this work is a modified version of the classical iterative Lucy-Richardson algorithm, in which we have implemented a smoothing operator that acts as a regularization scheme capable of recovering smooth and accurate distributions. The regularization depends on a single phase-dependent parameter (${\omega }_{p}$), which can be easily optimized without any significant user arbitrariness.

The algorithm performances have been tested by computer simulations, in which noisy in silico WAXTS-DSE data associated to samples made of a mixture of different TiO_2_ polymorphs NC and by microstrained iron telluride NC, have been inverted and the recovered distributions compared with the expected ones. In all the cases, the reconstruction of both the scattering data and the PSDs is highly accurate, also when the noise present in the data is much higher than typical levels achievable at synchrotron facilities. The algorithm features have been also discussed in comparison with the standard PSD analysis of the DEBUSSY Suite. Whereas our method works fairly well regardless of the PSD shapes and is capable of accurately recovering both number and mass PSDs, the DEBUSSY analysis becomes critical when the PSDs to be recovered are rather broad (${\sigma }_{n} / {\langle d\rangle }_{n}\ge 0.5$) and their shapes are fairly different from a LogNormal distribution (such as for a Weibull or an exponentially decaying PSD). In these cases, the DEBUSSY method recovers with a satisfactory accuracy only the mass PSD (accuracy on ${\langle d\rangle }_{m}$ and ${\sigma }_{m}$ of $\sim 5{-}10\%$), but wildly fails in recovering the number distribution with errors that are of $\sim 20\%$ for both ${\langle d\rangle }_{n}$ and ${\sigma }_{n}$ of the Weibull distribution and become $\sim 60\%$ for ${\langle d\rangle }_{n}$ of the Exponential distribution. Thus, any comparison between the DEBUSSY results and other techniques that work by analysing number PSDs (such as TEM or other optical microscopy methods) must be taken with high care. These findings also suggest that size values from a PSD function based on a wrong pre-assumption may result in similar inaccurate determinations.

When applied to the inversion on WAXTS data taken on real samples, the method relies on a (mandatory) accurate modelling of the nanoparticles crystal structure, which include local structural defects, microstrain, surface induced distortions, etc.. Indeed, such defects produce an additional (to the finite-size effect) broadening of the diffraction peaks that, if not properly taken into account in the NC modelling, might be misleadingly attributed to smaller NCs rather than to (defective) larger NCs. Thus, defects and sizes are correlated. Disentangling them can be a hard task, which, anyway, is an intrinsic problem common to any data analysis method. Similarly, it is mandatory to have accurate estimates of the atomic Debye–Waller and site occupancy factors. Fortunately, both factors can be estimated from literature or can be derived by a standard Rietveld analysis of the WAXTS data. Provided that all these requirements are fulfilled, the inversion algorithm works quite nicely also on real data, as demonstrated by the two examples reported in this work, namely a Magnetite-Maghemite nanopowder and a commercial P25-Titania sample composed by a mixture of anatase and rutile polymorphs. In both cases, the outcome of our inversions compares quite nicely with the results obtained by the DEBUSSY analysis.

We would like to emphasize that the inversion of WAXTS data (as well the DEBUSSY analysis) heavily relies on the use of the DSE, which is the theoretical tool providing the *entire* (peak and diffuse) and *exact* (including defectiveness or non-periodicity) scattering profile of the NCs. These features (not available with other conventional XRPD methods) are of fundamental importance for the correct functioning of the inversion algorithm, which requires both accurate modelling of the NCs structure and correct computing of the kernel functions over the entire Q-range of the measurement.

Finally, we would like to recall that the inversion method proposed in this work applies only to NCs described by monovariate distributions, i.e. NCs with spherical (or spheroidal) morphologies that require only one size parameter. This limitation currently hampers the applicability of the method to real anisotropic NCs but, as already mentioned in the text, in this work we considered only monovariate distributions as a proof of concept. Future work is indeed planned to extend the algorithm to bivariate distributions, aiming at characterizing truly anisotropic NCs in terms of their Particle Size and Shape Distributions (platelets or whiskers). Such bivariate distributions have indeed been experimentally observed on a number of technologically relevant nanomaterials (e.g. in perovskites^55^, biomimetic materials^31^, supported metals^28^) and derived by the DEBUSSY approach within the bivariate LogNormal assumption.

## Supplementary information

Supplementary Information 1.
