# Supplementary material for: Nanoparticle size distribution from inversion of wide angle X-ray total scattering data
Source: Sci Rep. 2020 Jul 29;10:12759. doi: 10.1038/s41598-020-69371-7 (PMC7391761; doi:10.1038/s41598-020-69371-7)
Supplement: Supplementary file 1 — Supplementary Information 1. [file 41598_2020_69371_MOESM1_ESM.pdf]

SUPPLEMENTARY INFORMATION

Nanoparticle size distribution from inversion of Wide Angle X-ray Total Scattering data

Fabio Ferri, Federica Bertolotti, Antonietta Guagliardi, and Norberto Masciocchi

SUMMARY

1) **Inversion Algorithm: optimization of the smoothing parameter ω_p.**

2) **Inversion Algorithm: stopping criterion.**

3) **Simulations: number distributions.**

4) **Inversion Algorithm: stability against noise.**

5) **Inversion algorithm: artefacts in the recovered distributions deriving from imperfect modeling.**

6) **Inversion algorithm: comparison with DEBUSSY analysis.**

7) **Synchrotron WAXTS data collection and reduction.**

8) **Modeling and scattering profiles of Magnetite-maghemite (Fe_3O_4 - γ-Fe_2O_3) nanocrystals.**

9) **Modeling and scattering profiles of commercial Titania (TiO_2) nanocrystals.**

10) **Inversion from multimodal distributions.**

11) **Ill-posedness analysis of the WAXTS-DSE data inversion problem.**

1) Inversion Algorithm: optimization of the smoothing parameter ω_p

As explained in the main text, the novelty of our algorithm, with respect to the original Lucy-Richardson algorithm, is the introduction of a smoothing procedure of the recovered distributions between successive iterations. This procedure consists in convolving the recovered distribution (for each phase *p*) with a 3-points symmetric triangular operator Λ_{ω_p}, in which the amplitudes of the lateral points are set by the parameter ω_p. Such a convolution acts as a regularization scheme that produces smooth distributions, but the price to pay for such regularization is a dampening of the ultimate resolution of the method. Indeed, the higher ω_p, the broader the distribution that can be reliably recovered. In other words, narrow distributions are artificially broadened if a too large value for ω_p is used, dampening in this way the resolving power of the method.

An example of these effects is shown in Figure 1, where the noisy data ($SNR\sim300$) deriving from two Log-Normal distributions of anatase TiO_2 nanocrystals with the same (weight) average diameter $< d_1 >_m = < d_2 >_m = 15.6nm$, but quite different widths $\sigma_1 = 6.2nm$ (Fig.S1(a)) and $\sigma_2 = 0.50nm$ [Fig.S1(a)], were inverted with different values of ω_p (including $\omega_p = 0$) varying in the range $10^{-6} - 10^{-1}$. As one can immediately notice, the recovered distributions of Fig.S1(a) are fairly dependent on ω_p, passing from highly spiked curves at small ω_p to nicely smoothed curves for large ω_p's where, however, the reconstruction is not so accurate. In between, there is an optimal value of $\omega_p\sim10^{-3}$ for which the matching between the recovered and the input distribution is reasonably good, as also witnessed by the residual plots reported in Fig.S1(b). When the input distribution is quite narrow as in Fig.S1(c), the optimal ω_p value tend to be $\omega_p\sim0$ and as larger and larger ω_p's are used, the recovered distribution becomes increasingly over-smoothed, spoiling the resolution of the inversion algorithm. Thus, it is quite evident that, for this simulation, the optimal value of ω_p is highly dependent on distribution width and must be optimized.

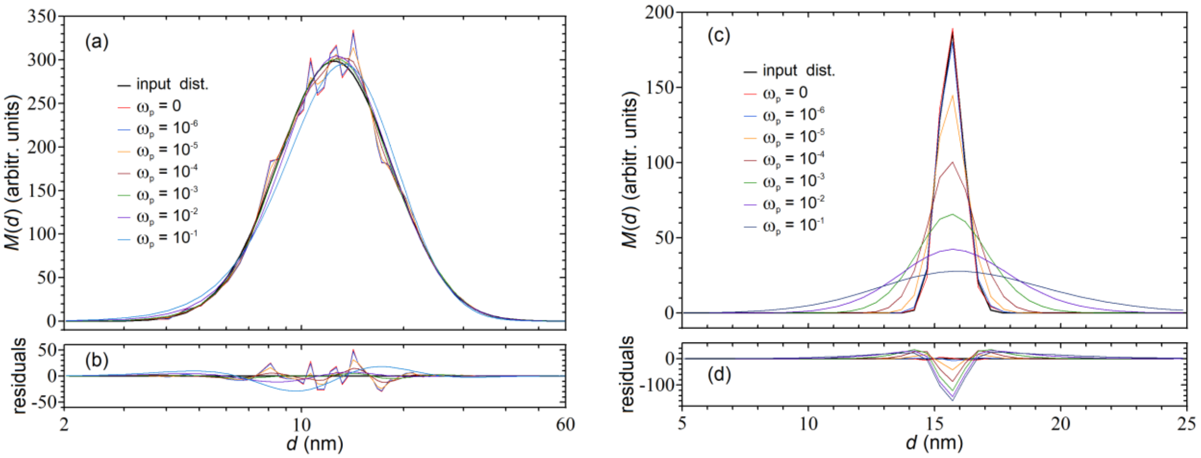

Figure S1 – (a) Simulated input (black curve) and reconstructed (colour curves) mass distributions obtained at different values of the smoothing parameter ω_p for anatase TiO_2 nanocrystals characterized by a fairly broad Log-Normal distribution ($< d_1 >_m = 15.6nm$, $\sigma_{1m} = 6.2nm$); (b) Absolute residuals between recovered and input distributions of panel (a); (c) same as a panel (a), but with a very narrow Log-Normal distribution ($< d_2 >_m = 15.6nm$, $\sigma_{2m} = 0.5nm$); (d) Absolute residuals between recovered and input distributions of panel (c).

Another factor which is expected to influence the optimal value of ω_p to be used in the inversion procedure is the actual noise present in the data. To quantitatively investigate this effect, we repeated the same simulations of Fig.S1(a) and Fig.S1(b) by generating independent noisy WAXTS-DSE signals, all of them characterized by the same level of statistical Poisson noise ($SNR{\sim}300$), which is equal to the typical noise encountered in total scattering experiments of solid (*i.e.* dry) nanoparticles performed at dedicated synchrotron beamlines. Each input signal was inverted with different values of ω_p and the inversion procedure was stopped as described below in the second paragraph of this SI. The agreement between the distributions recovered at the different ω_p and the input one, was ascertained by using the Relative Restoration Error (RRE) parameter defined as

$$\mathrm{RRE}(\omega_p) = \frac{\sum_{j=1}^{M}\left(M_j^{rec} - M_j^{inp}\right)^2}{\sum_{j=1}^{M}\left(M_j^{inp}\right)^2} \qquad \text{(S1)}$$

where the mass distributions are computed as $M(d_j) = N(d_j)\, m_j$ (note that the phase index *p* has been omitted for simplicity). Eq.(S1) corresponds to the relative average mean square deviations between the retrieved and input mass distribution. Thus, the optimal value of ω_p is, in principle, the one that minimizes RRE.

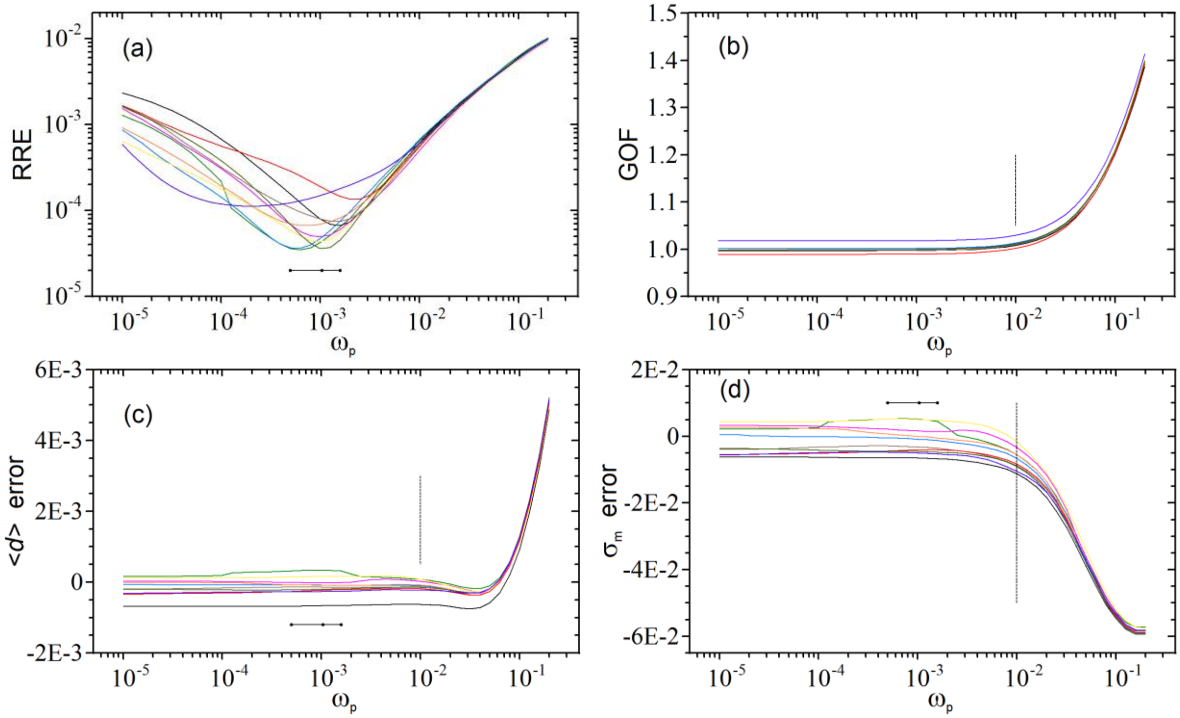

Figure S2 – Behaviors, as a function of ω_p, of various parameters characterizing the distribution of Fig.S1(a) for 10 different configurations of statistical Poisson noise of the same level ($SNR = 300$). (a): Relative Reconstruction Error (RRE) between the input and recovered distributions; each curve exhibits a minimum at ω_p^* whose average value is $\langle\omega_p^*\rangle = 1.04 \times 10^{-3} \pm 5.4 \times 10^{-4}$ (see horizontal bar). (b) Goodness of Fit (GOF) parameter (see Eq.5 main text); (c) Relative error $[(rec\text{-}inp)/inp]$ between the recovered and input average diameters. (d) Relative error between the recovered and input standard deviations. The horizontal bar indicates the range $\langle\omega_p^*\rangle \pm \sigma_{\omega_p}$. Vertical bar in (b) indicates the value $\omega_p{\sim}10\,\langle\omega_p^*\rangle$ at which the GOF starts to deviate from 1. Vertical bars in (c) and (d) show that both errors start to deviate from 0 at values well beyond $\omega_p{\sim}10\,\langle\omega_p^*\rangle$.

In Fig.S2(a) we report the behavior of RRE as a function of ω_p for the same distribution of Fig.S1(a) corresponding to 10 statistically independent configurations of Poisson noise. As one can notice, the curves change remarkably for different configurations and exhibit minima, ω_p^*, that are very broad and spread over a pretty large range of ω_p's. Their average value is $\langle\omega_p^*\rangle = 1.04 \times 10^{-3} \pm 5.4 \times 10^{-4}$. In Fig.S2(b) we report the behavior of GOF as a function of ω_p corresponding to the same curves shown in Fig.S2(a). This figure suggests that there is an extremely large range of ω_p's (from $\omega_p \ll \langle\omega_p^*\rangle$ to $\omega_p \sim 10\,\langle\omega_p^*\rangle$, indicated by the vertical bar) where, regardless of the fact that RRE might be quite higher than its minimum value, the signal reconstruction is always excellent with values of GOF~1. Similarly, within this range, the distribution recovery is always accurate, as evidenced by Fig.S2(c) and S2(d), where the relative errors [(*rec.-inp*)/*inp*] between the recovered and input average diameters and standard deviations are reported as a function of ω_p. Both figures exhibit the same behaviors, showing that, as long as $\omega_p \leq 10\,\langle\omega_p^*\rangle$, both parameters are recovered quite accurately, with relative errors always smaller than $\sim 0.1\%$ (average diameter) and $\sim 1\%$ (standard deviation).

Figure S3 reports the same analysis of Fig.S2 for the distribution of Fig.S1(b), which is much narrower. In this case, the minima ω_p^* are even more scattered and, on average, are much smaller, *i.e.* $\langle\omega_p^*\rangle = 3.2 \times 10^{-7} \pm 3.2 \times 10^{-7}$ Similarly to Fig.S2, as long as $\omega_p \leq 10\,\langle\omega_p^*\rangle$ (vertical bar), GOF~1 and the relative errors on the average diameter remain always smaller than $\sim 0.1\%$. As to the standard deviation, Fig.S3(d) suggests that the optimal value of $\omega_p \rightarrow 0$, but also in this limit the errors on σ_m remain always

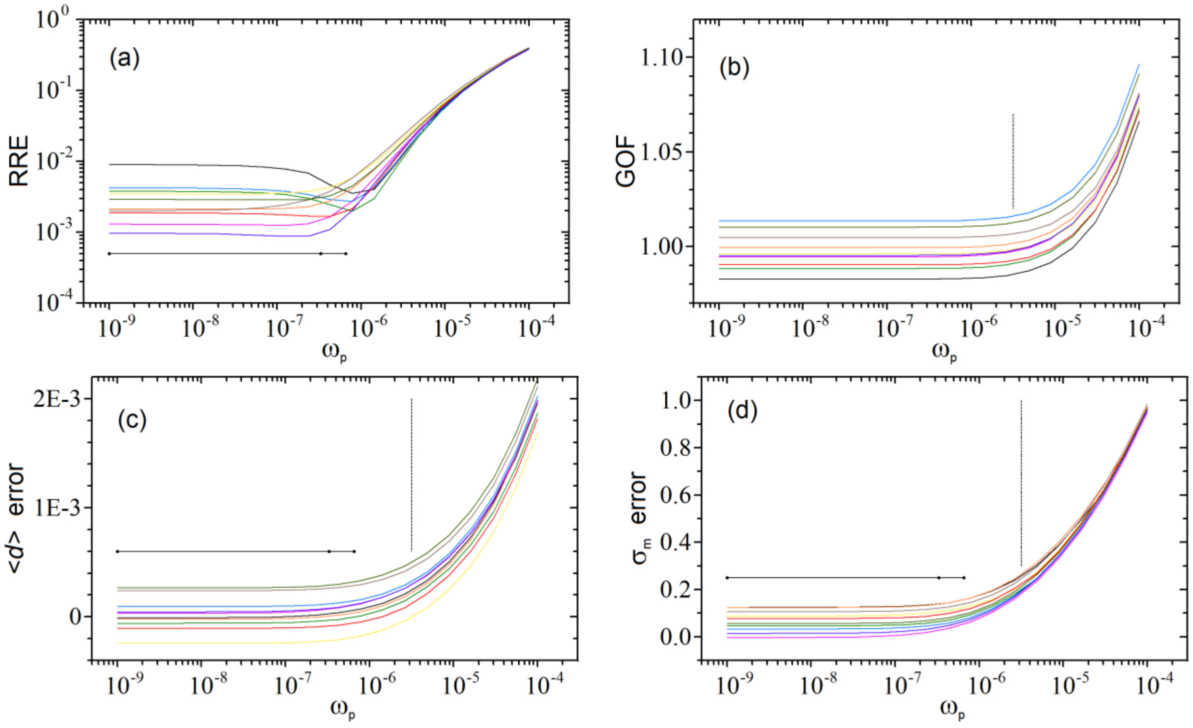

Figure S3 – Behaviors, as a function of ω_p, of various parameters characterizing the distribution of Fig.S1(b) for 10 different configurations of statistical Poisson noise of the same level ($SNR = 300$). (a): Relative Reconstruction Error (RRE) between the input and recovered distributions; each curve exhibits a minimum at ω_p^* whose average value is $\langle\omega_p^*\rangle = 3.2 \times 10^{-7} \pm 3.2 \times 10^{-7}$ (see horizontal bar). (b) Goodness of Fit (GOF) parameter (see Eq.5 main text); (c) Relative error [$(rec\text{-}inp)/inp$] between the recovered and input average diameters. (d) Relative error between the recovered and input standard deviations. The horizontal bar indicates the range $\langle\omega_p^*\rangle \pm \sigma_{\omega_p}$. Vertical bar in (b) indicates the value $\omega_p \sim 10\,\langle\omega_p^*\rangle$ at which the GOF starts to deviate from 1. Vertical bars in (c) and (d) show that both errors start to deviate from 0 at values well beyond $\omega_p \sim 10\,\langle\omega_p^*\rangle$.

$\sim 10\%$, a limitation due to the intrinsic finite resolution of the inversion procedure. On the other side, for values of $\omega_p > \langle\omega_p^*\rangle$, the figure shows that, up to $\omega_p \sim 5\,\langle\omega_p^*\rangle$, the errors on σ_m are always $\leq 20\%$.

Summarizing, Figs.2 and 3 show that, although the data associated to each single noise configuration should be inverted with their own optimal ω_p^*, if we use a unique single optimal $\omega_p^{opt} = \langle\omega_p^*\rangle$, the errors introduced in the recovery of the distribution parameters are quite negligible (except for the parameter σ_m of the narrow distribution, which remain always $\sim 10\%$, due to the intrinsic finite resolution of the procedure).

Clearly, we expect that ω_p^{opt} is also dependent on many other parameters such as distribution shapes, crystal phases and the absolute level on noise present on the data. For this reason, we repeated the same test of Figs.2 and 3 with different levels of Poisson noise, by using crystals of anatase and rutile, characterized by Log-Normal and Gaussians distributions with various average diameters and standard deviations. Our findings show that, regardless of all the other parameters, ω_p^{opt} is *mainly* dependent on only two parameters: the width σ_m of the (mass) recovered distribution and the noise level present in the data. Figure 4 reports the behavior of ω_p^{opt} (symbols) as a function of σ_m for a variety of different distributions and four noise levels ($SNR = 30, 100, 300, 1000$). As one can notice, for each σ_m, the values of ω_p^{opt} span over about two decade, passing from $\omega_p^{opt} \sim 10^{-7} - 10^{-5}$ for very narrow distributions ($\sigma_m \sim 0.5 - 1nm$) up to values which tend to saturate around $\omega_p^{opt} \sim 10^{-3} - 10^{-1}$ for broad distributions with $\sigma_m \geq 10nm$. This range of variability of about two decade is mostly due to different noise levels, with noisiest data ($SNR = 30$) requiring values of $\omega_p^{opt} \sim 100$ times higher than those required for least noisy data ($SNR = 1000$).

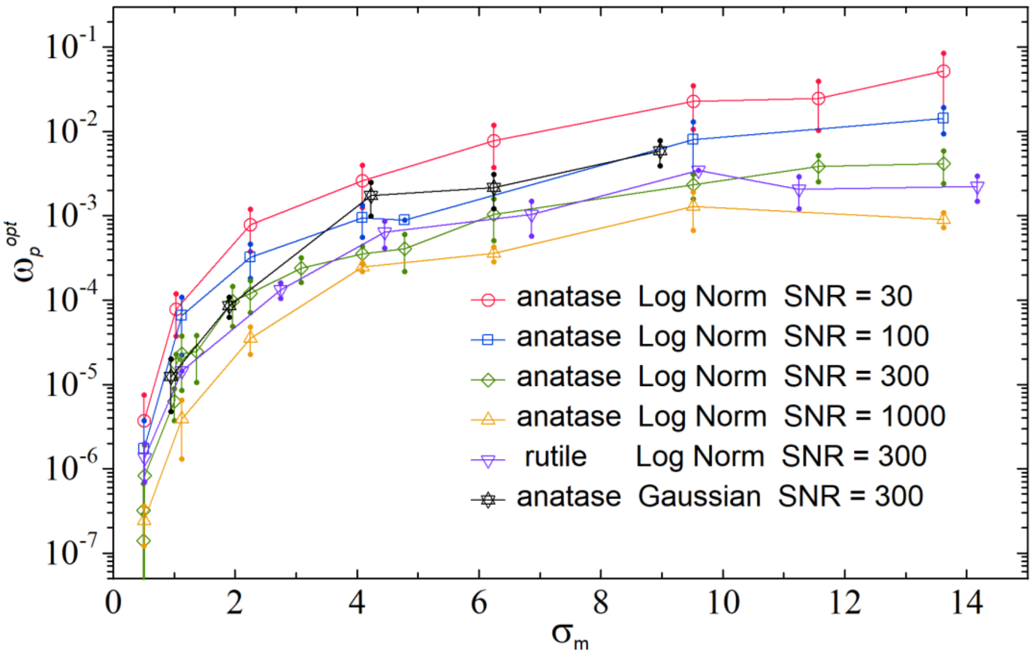

Figure S4 – Behaviors, as a function of σ_m, of the optimal smoothing parameter ω_p^{opt} for a variety of different distributions and four noise levels. The lines are guide to the eye.

In conclusion, Fig.S4 represents a clear indication for the choice of ω_p when σ_m and *SNR* are known. However, when these two parameters are not (or only roughly) known prior the inversion, a rule of thumb for choosing ω_p is the following:

(a) carry out the inversion with $\omega_p = 0$;
(b) estimate σ_m [which is not affected by $\omega_p \to 0$ as shown in Figs.S2(d) and S3(d)];
(c) by using Fig.S4, choose a mid-range value for ω_p corresponding to σ_m;
(d) repeat the inversion with the new value of ω_p and compute the new σ'_m;
(e) if $\sigma'_m > \sigma_m$ decrease ω_p(for example by a factor 2) and go back to point (d);
(f) if $\sigma'_m \sim \sigma_m$ and the recovered distribution is still too spiky (see below), increase ω_p(for example by a factor 2) and go back to point (d);
(g) if $\sigma'_m \sim \sigma_m$ and the recovered distribution is sufficiently smooth (see below), accept the result and the procedure is over.

As a final comment about the spikiness (or smoothness) of the recovered distribution, we found that, although possible, a quantification of this feature was not necessary. Indeed, as long as $\omega_p < \omega_p^{opt}$(see panels (c) and (d) of Figs.S2 and S3), both $< d >_m$ and σ_m are unaffected by ω_p.Thus, distributions with different levels of spikiness provide the same recovered parameters and selecting the one which is sufficiently smooth is a simple criterion of "good sense" based on a visual inspection of the curve.

2) Inversion Algorithm: stopping criterion

The iterative procedure was stopped according to the following criteria:

(*a*) First of all, we impose a minimum number of iterations, r_{min}, which is necessary for the algorithm to work properly, i.e. to reconstruct accurately the expected distribution under ideal conditions (noiseless data). This is necessary because the starting uniform distribution is (obviously) very different from the expected one and the LR algorithm attains convergence quite slowly. The parameter r_{min} was estimated by finding the number of iterations necessary for retrieving the expected (mass) distributions with high accuracy (RRE $\sim 10^{-3}$). We tested many distributions of a single phase (anatase) TiO_2 nanocrystals with different shapes (Log-Normals and Gaussians) and different average diameters and standard deviations. All the inversion were carried out by imposing $\omega_p = 0$, which is the optimal value for noiseless data. The results indicate that, regardless of average diameters and distribution shapes, r_{min} is mainly dependent on the (mass) standard deviations the input distributions as described in Table S1.

TABLE S1: minimum number of iterations r_{min} used for inverting a single phase (anatase) TiO_2 nanocrystals

σ_m (nm)	r_{min}
1	$4\times10^4 - 7\times10^4$
2	$8\times10^3 - 2\times10^4$
5	$3\times10^3 - 7\times10^3$
10	$2\times10^3 - 5\times10^3$
15	$1\times10^3 - 3\times10^3$

Notice that r_{min} scales approximately as the inverse of the distribution width ($r_{min} \propto 1/\sigma_m$) and for the narrowest distributions ($\sigma_m = 1$ nm) tends to be rather high ($> 10^4$).

(*b*) We also impose a maximum number of iterations $r_{max} = 10^6$, which ensures that the inversion stops even when the criteria below reported are not met.

(c) For any $r_{min} < r < r_{max}$, the procedure is stopped as soon as the parameter GOF(*r*), computed for each iteration, attains a minimum and continues to increase for at least 10 consecutive iterations.

(d) Additionally, whenever, for $r_{min} < r < r_{max}$, condition (*c*) is not met but the decrease of GOF(*r*) becomes increasingly slow, the procedure is stopped when the variation of GOF(*r*) is below a given threshold. Numerically, we monitored the parameter $\delta(r) = [d\ \mathrm{GOF(r)/dr}]/\mathrm{GOF(r)}$ (equal to the (relative) first derivative of GOF with respect to *r*) and stopped the procedure when $\delta(r) \leq 10^{-9}$.

Finally, we checked that, when the procedure would prefer to stop at a number of iterations $r < r_{min}$ either because conditions (c) or (d) are met, forcing it to continue up to r_{min} does not jeopardize significantly the quality of the recovered distribution.

3) Simulations: number distributions

Table S2 reports the comparison between the *number* input and recovered distribution parameters relative to the TiO_2 simulation described in Fig.2 of the main text.

TABLE S2: comparison between input and recovered parameters for the *number* distributions of Fig.2 of the main text

	input			recovered		
TiO_2 phase	<*d*>$_n$ (nm)	σ_n (nm)	c_n (%)	<*d*>$_n$ (nm)	σ_n (nm)	c_n (%)
anatase	4	1	0.625	3.87	1.12	0.632
rutile	6	2	0.250	5.98	2.03	0.242
brookite	10	2	0.125	9.73	2.31	0.126
background (a.u.)	--	--	1	--	--	0.998

Table S3 reports the comparison between the *number* input and recovered distribution parameters relative to the Fe_5Te_4 simulation described in Fig.3 of the main text.

TABLE S3: comparison between input and recovered parameters for the *number* distributions of Fig.3 of the main text

	input			recovered		
Fe_5Te_4 phase	<*d*>$_n$ (nm)	σ_n (nm)	c_n (%)	<*d*>$_n$ (nm)	σ_n (nm)	c_n (%)
no-strain	-	--	0	5.28	3.30	0.003
aniso-strain	10	2	1	9.85	2.21	0.980
iso-strain	--	--	0	8.28	3.00	0.017
background (a.u.)	--	--	1	--	--	0.998

4) Inversion Algorithm: stability against noise

One of the main advantages of having introduced our smoothing procedure in the original LR algorithm is the remarkable stability of our algorithm against noise. Indeed, whereas for the original LR procedure the recovered distributions tend to become spiky when too many iterations are processed, in our case, they always remain nicely smooth without renouncing to accuracy (provided that ω_p is properly chosen). We have ascertained the stability of our algorithm against noise by repeating the same simulation test of Fig.2 of the main text (a TiO_2 sample made of a mixture of nanocrystals of the three common polymorphs *rutile*, *anatase* and *brookite*) with three different noise levels corresponding to $SNR \sim 300, 100, 30$.

For each of them, we accumulated statistics by generating (and inverting) 100 independent noisy $I(Q)$ scattering profiles obtained by adding 100 statistical independent configurations of Poisson noise to the ideal (noiseless) profile. Then we evaluated the algorithm performances in terms of accuracy on the recovered distribution parameter. The results of this test are shown in Figs.5 and 6.

Figure 5(a) compares the input distributions of the three phases (black solid curves) with the corresponding recovered *averaged* distributions (colored symbols) obtained by averaging 100 distributions retrieved by inverting 100 noisy data ($SNR \sim 100$). The error bars associated to each point of the recovered distributions are remarkably small (not visible on the scale of the figure), demonstrating the high stability of the inversion procedure. At the same time the matching between the input and recovered distribution is rather good, as demonstrated by the normalized residual plots ($norm\_res_i = (M_i^{rec} - M_i^{inp}) / \sum_i M_i^{inp}$) reported in Fig.S5(b), where the (systematic) errors are always smaller than 1%. On the right side of the figure, we report for all the three noise levels, as a function of the noise configuration number, the GOF (c), the stopping iteration number (d), and the relative error on the recovered background amplitude (e). As one can notice, the GOF is always around unity (± 0.02), the number of iterations varies between $\sim 1 - 5 \times 10^4$, and the relative error on the background amplitude is always lower than $\sim 1\%$.

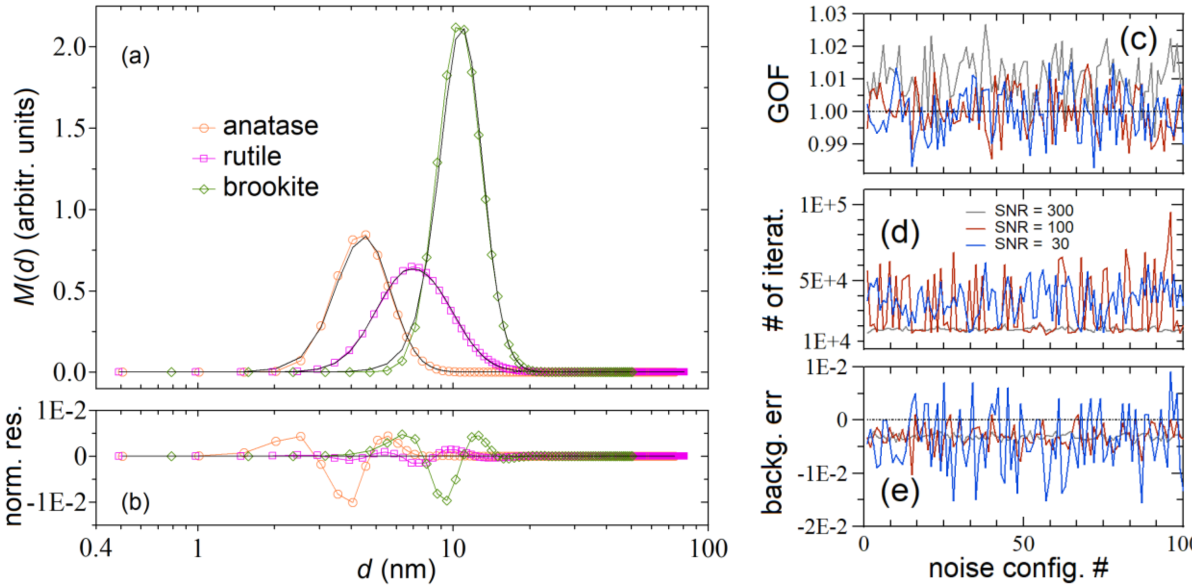

Figure S5 – (a) Simulated input (black curves) and average recovered (colour curves) mass distributions of the three phases of a TiO_2 sample made of a mixture of anatase, rutile and brookite. The distribution parameters and the noise level on the data are the same of Fig.2 of the main test. The average distribution and error bars (not visible because very small) were computed by averaging 100 distributions retrieved by inverting 100 independent noisy data with SNR=100. (b) normalized residuals between the recovered and input distributions. (c-d-e) behaviours, as a function of SNR and noise configuration number, of GOF (c), stopping iteration number (d), and relative error on the recovered background amplitude (e).

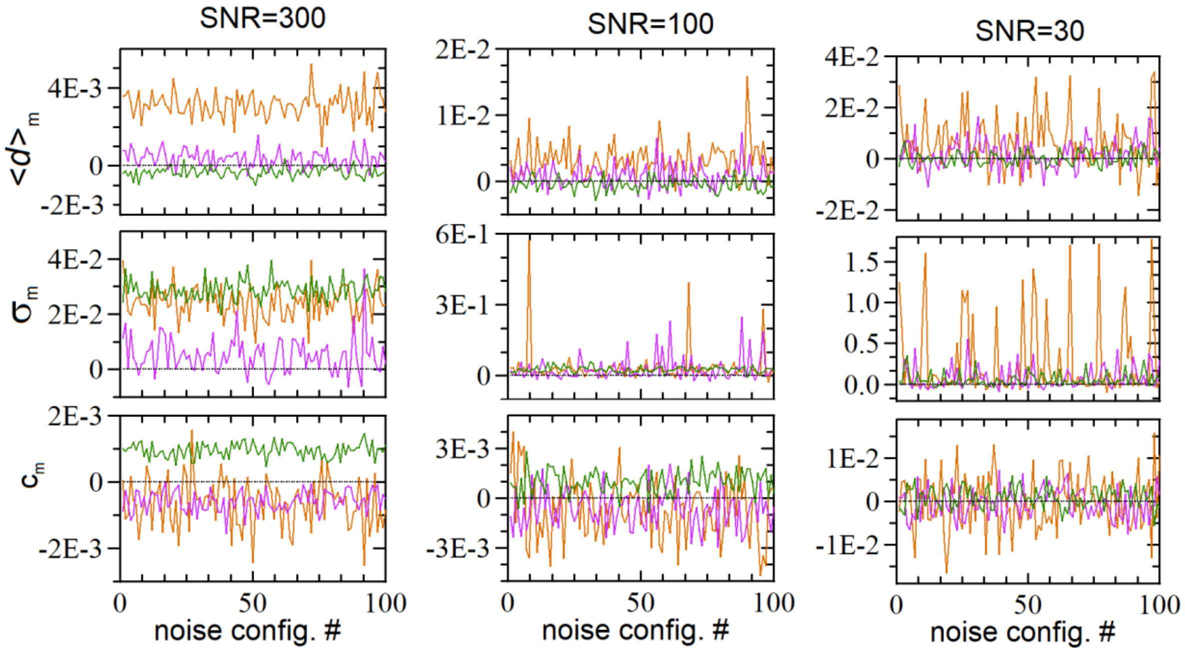

Figure S6 – Behaviours, as a function of SNR and noise configuration number, of the relative errors between the recovered and input parameters for the three phases anatase (orange), rutile (magenta) and brookite (green) of Fig5: $< d >_m$ (first row), standard deviation σ_m (second row) and concentration c_m (third row)

Figure 6 reports for all the three noise levels, as a function of the noise configuration number and for all the three phases, the relative errors between the recovered and expected (mass) average diameter $< d >_m$ (first row), standard deviation σ_m (second row) and concentration c_m (third row). As expected, the errors increase with the noise level (lower SNRs), but for common experimental conditions where typically $SNR \geq 100$, they are always much less than 1% for $< d >_m$ and c_m, and less than a few percents for σ_m.

Therefore, we can conclude that the stability of our algorithm against noise is remarkably high.

5) Inversion algorithm: artefacts in the recovered distributions deriving from imperfect modeling

In this section we report some examples of the artefacts that might arise in the recovered distribution when there is some discrepancy between the modelled and actual structure of the nanocrystals or there are some errors in the (shape of the) background signal. We anticipate that these inaccuracies introduce systematic errors in the kernel functions that show up as spurious peaks in the recovered distributions.

Figure 7 shows an example of what happens when the background signal is not properly taken into account. The input data have been generated by summing the WAXTS-DSE data (Fig.S7(b), black curve) corresponding to a distribution of anatase TiO_2 nanocrystals (Log Normal, $< d >_m = 24nm$, $\sigma_m = 6.0nm$) to a linear background (blue curve). The latter one is quite small with respect to the scattering peaks, but not so small with respect to the diffuse scattering in between the peaks. The inversion has been carried out by using either no background or a constant background. The corresponding recovered mass distributions (colored symbols) are shown in Fig.S7(a), together with the input distribution (black solid curve). As evident, the recovered distributions match the input one rather accurately over almost the entire diameter range, except for the very narrow sizes where two spurious peaks are present. These peaks are due to the fact that, since the background is not available or not properly shaped for the signal reconstruction, this missing contribution is attributed to the smaller sizes that are the ones whose scattering profiles resemble more closely the background signal (see magenta curve in Fig.S6(b), which corresponds to the second lowest size used in the nanocrystal distribution). It is worth noticing that the two spurious peaks are very well separated from the main bell shaped distribution; thus, they can be easily trimmed out when computing the distribution average parameters. For completeness, we report in Fig.S7(c) also the input and recovered number distributions, in which the spurious peaks are enormously amplified. Nevertheless, as for the mass distributions, they are still well isolated and can be easily removed from the analysis.

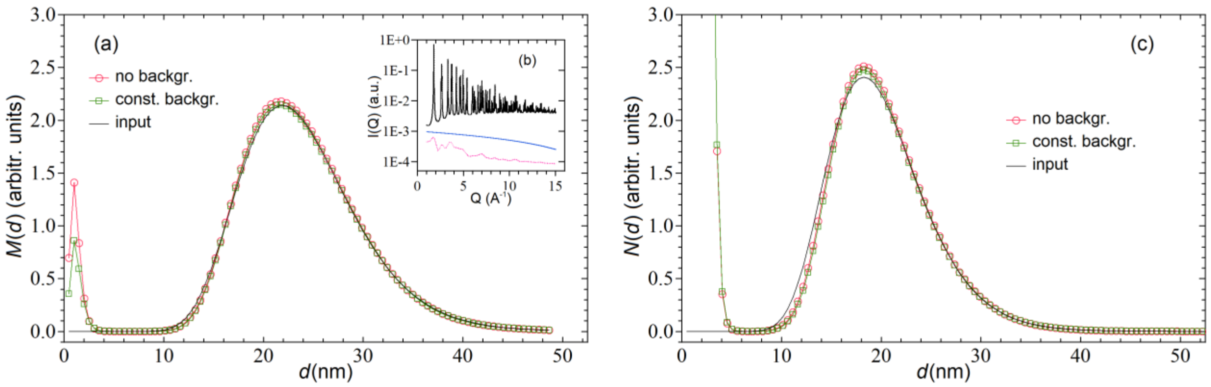

Figure S7 – (a) Simulated input (black curve) and recovered (colour symbols) *mass* distributions of a TiO_2 sample (see text) when the inversion is carried out by using an incorrect constant background signal (green squares) or no background (red circles); (b) WAXTS-DSE data (black curve) and corresponding background signal (blue curve) used for generating the input data. For comparison with the background signal, the scattering profile of the second lowest size of the distribution has also been reported (magenta dotted curve); (c) *number* distributions corresponding to the mass distributions of (a).

The second example shows what happens when the Debye – Waller thermal parameters T_i are not correct. Figure 8a compares the input distribution of anatase TiO_2 nanocristyals (Log Normal, $< d >_m = 12nm$, $\sigma_m = 4.4nm$) with the distributions recovered when the input data have been generated with $T_{inp} = 0.7$ Å, but inverted with $T_{rec} = 0.5$ Å (red circles) and vice versa (green squares). The effects of varying T_{inp} between 0.5 Å and 0.7 Å is shown in the figure inset [Fig.S8(b)], where is quite evident that the main difference between the two profiles shows up in the diffuse scattering between the peaks. In these regions, the sample with higher T_{inp} scatters slightly more ($\sim 10\%$) than the sample with smaller T_{inp}. Thus, the situation is similar to that discussed in Fig.S7, where an incorrect background signal was used. It is indeed well known that thermal factors and the background level, even in conventional powder diffractometry, highly correlate, and manifest themselves in additional scattering, attributable to diffuse or extra-sample contributions, respectively. Correspondingly, the distribution of Fig.S8(a), which was recovered by using kernel functions with not enough scattering in between the peaks (very much as in the case of an underestimated background level) shows a spurious peak at small sizes (red circles). On the contrary, had the input data generated with $T_{inp} = 0.5$ Å, and inverted with $T_{rec} = 0.7$ Å, an excess diffuse scattering appears, which can be dampened only by reducing the population of the smallest classes belonging to the input distribution (green squares). In the latter case the occurrence of this artefact is less evident, and care must be taken into interpreting the inversion results. For completeness, we report in Fig.S8(c) also the input and recovered number distributions, in which the two artefact are remarkably amplified with respect to the ones shown in the mass distributions. Thus, the recovery of number distributions in the presence of this kind of artefacts becomes highly critical.

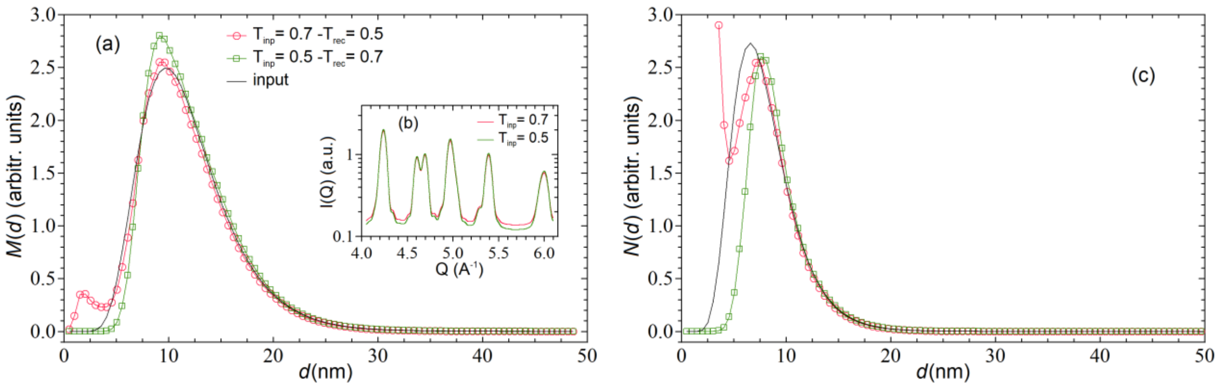

Figure S8 – (a) Simulated input (black curves) and recovered (colour symbols) *mass* distributions of the anatase phase of a TiO2 sample when the inversion is carried out by using incorrect Debye – Waller thermal parameters, i.e. different from the ones used for generating the scattering data; (b) detail of the WAXTS-DSE data generated by using $T_{inp} = 0.7$ Å (red curve) and $T_{inp} = 0.5$ Å (green curve); ; (c) *number* distributions corresponding to the mass distributions of (a).

6) Inversion algorithm: comparison with DEBUSSY analysis.

In this section we report two examples concerning the comparison between our algorithm and the DEBUSSY analysis. As known, the DEBUSSY method pivots on the strong assumption that the PSD of the sample is supposed to be known (typically a LogNormal distribution) and the various distribution parameters (average size, standard deviation, Debye-Waller thermal parameters, background amplitude, etc.) are retrieved by standard X^2 minimization. Thus, in those cases where the PSD shapes are fairly different from a LogNormal, this wrong assumption could affect significantly the results, and the fitted distribution parameters might be highly inaccurate.

In Fig.9 we report this comparison for the case of a Weibull (number) input distribution of anatase TiO_2 nanocrystals with $\langle d \rangle_n = 10nm$ and $\sigma_n = 5nm$. Figure 9(a) and (b) compare the input number and mass distributions (black solid curves) with the corresponding *averaged* distributions recovered with the DEBUSSY analysis (blue squares) and with our algorithm (red circles). Statistics was accumulated by averaging 100 distributions retrieved by processing 100 noisy data generated with $SNR = 300$. As one can notice, the error bars are always remarkably small (not visible on the scale of the figure) except for the small sizes of the number distributions recovered with our algorithm (where, anyway, there is statistical consistency between input and recovered distributions). Thus, Figures 9(a) and 9(b) show that, whereas our algorithm is capable of retrieving both number and mass distributions with high accuracy, the DEBUSSY method recovers with a somewhat accuracy only the mass distribution, but wildly fails in the reconstruction of for the number distribution. The different performances of the two methods are also witnessed by the different qualities of signal reconstruction: with our algorithm we obtain a GOF $\sim 1.00 \pm 0.01$ whereas with the Debussy analysis we get GOF $\sim 3.16 \pm 0.02$. Although such differences are not appreciable in Fig.9(c) where both reconstructed signals are indistinguishable from the input signal (black circles), the residuals plot clearly shows that the DEBUSSY reconstruction (blue curve) exhibits systematic deviations that, in correspondence of the peaks, are much higher than the (non systematic)

deviations associated to our method (red curve). A summary of the results of this test together with more tests carried out with $SNR = 100$ and $SNR = 30$ are reported in Table S4. Regardless of the SNR, our

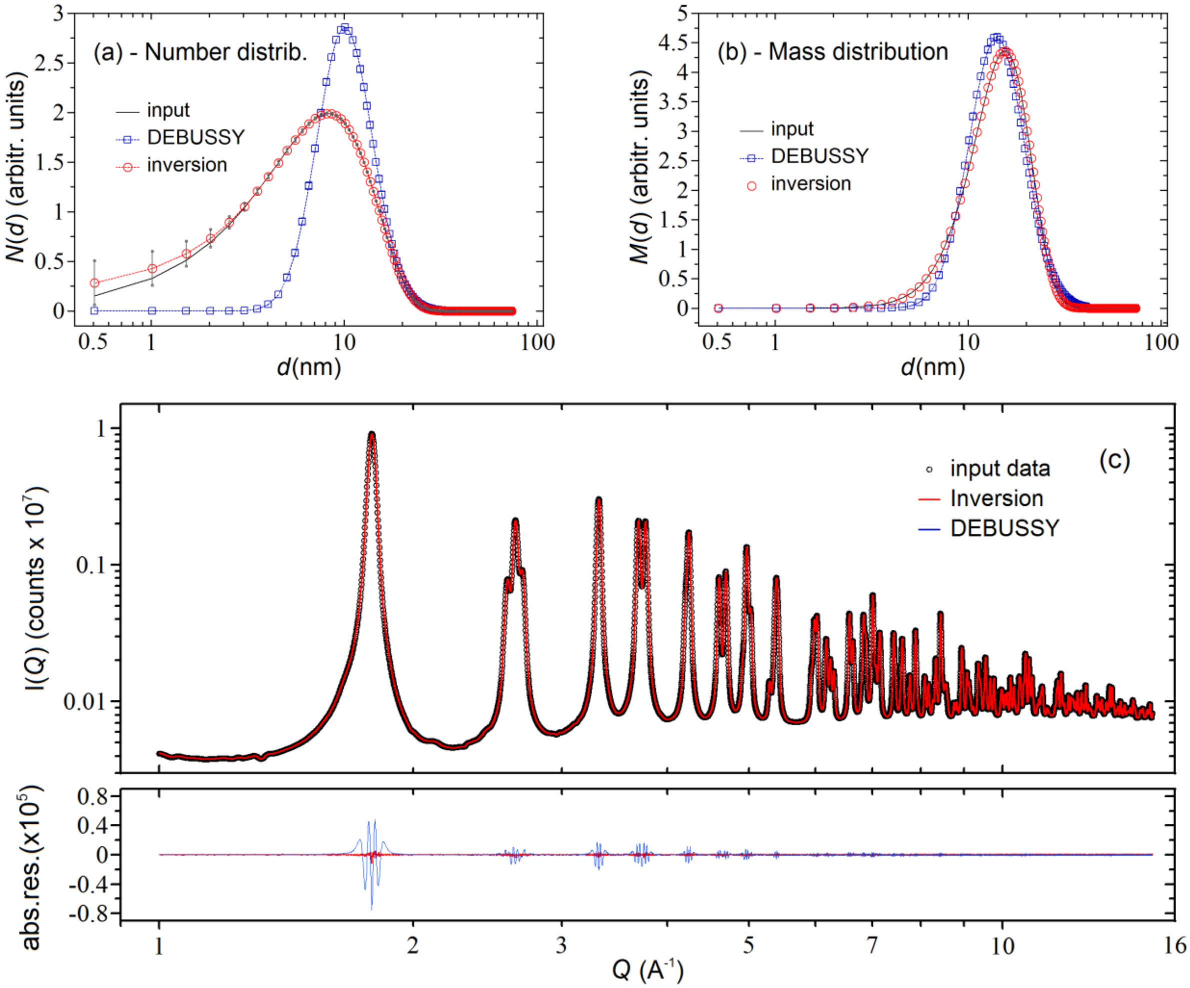

Figure S9 – (a) Simulated Weibull *number* input distribution of anatase TiO_2 nanocrystals with nominal $\langle d \rangle_n = 10nm$ and $\sigma_n = 5nm$ and corresponding (averaged) recovered distributions obtained with the DEBUSSY analysis (blue squares) and with our inversion algorithm (red circles). Statistics was accumulated by processing 100 noisy data with $SNR \sim 300$.; (b) corresponding input and recovered *mass* distributions; (c) Simulated input WAXTS (black circles) and reconstructed data obtained with the DEBUSSY analysis (blue line, not visible)and with our inversion algorithm (red line); (d) absolute residuals (recovered-input) for the data of panel c. DEBUSSY residuals are systematic and much higher than inversion residuals.

method recovers both number ($\langle d \rangle_n$, σ_n) and mass ($\langle d \rangle_m$, σ_m) parameters that are always consistent (within the statistical accuracy) with the input ones. Conversely, for the DEBUSSY analysis, $\langle d \rangle_n$ and σ_n are both wrong by $\sim 20\%$ whereas the mass parameters are somewhat more accurate with only σ_m off by $\sim 8\%$. It is also worth noticing that the GOF recovered with our method is always around unity, whereas for DEBUSSY depends sensitively on the SNR levels. As expected at low $SNR = 30$, the GOF is around unity because the systematic deviations between input and reconstructed data are smaller than noise (data not shown), but at high $SNR = 300$, the opposite takes place (see Fig.9d) and we get GOF $\sim 3.16 \pm 0.02$.

TABLE S4: comparison between input parameters of the Weibull *number* distribution of Fig.9 and the parameters recovered by using our inversion method and the DEBUSSY analysis. Three different SNR levels are shown.

	$<d>_n$ (nm)	σ_n (nm)	$<d>_m$ (nm)	σ_m (nm)	GOF
Input	10	5	16.360	5.122	
Inversion (SNR = 300)	9.94 ± 0.11	5.03 ± 0.06	16.365 ± 0.001	5.137 ± 0.003	1.00 ± 0.01
DEBUSSY (SNR = 300)	11.90 ± 0.02	4.05 ± 0.01	16.510 ± 0.005	5.52 ± 0.02	3.16 ± 0.02
Inversion (SNR = 100)	9.83 ± 0.16	5.08 ± 0.08	16.388 ± 0.003	5.20 ± 0.01	1.00 ± 0.01
DEBUSSY (SNR = 100)	11.892 ± 0.003	4.057 ± 0.001	16.510 ± 0.003	5.52 ± 0.01	1.38 ± 0.01
Inversion (SNR = 30)	9.6 ± 0.9	5.15 ± 0.3	16.373 ± 0.009	5.17 ± 0.03	1.00 ± 0.01
DEBUSSY (SNR = 30)	11.89 ± 0.02	4.06 ± 0.01	16.510 ± 0.01	5.53 ± 0.02	1.04 ± 0.01

The second test refers to an exponential decay (number) input distribution of anatase TiO_2 nanocrystals with nominal $\langle d \rangle_n = \sigma_n = 5\ nm$. As above, we processed 100 noisy input data signals with different SNR levels and, for $SNR = 300$, we obtained the results reported in Fig.10. Once again the distributions obtained with the DEBUSSY analysis are partially (mass) and highly (number) inaccurate, whereas the ones obtained with our algorithm match quite nicely the expected ones (both number and mass). The differences in signal reconstruction are very similar to the ones obtained in the previous case, with similar systematic residuals [see Fig.10(d)] and a higher GOF. A summary of the results of this test carried out also with $SNR = 100$ and $SNR = 30$ are are reported in Table S5. All the comments done for Table S4 apply also to Table S5.

In conclusion, we have shown two examples (a Weibull and an Exponential distribution) of the different performances between our inversion algorithm and the DEBUSSY analysis. In both cases the number distributions to be recovered were quite broad (σ_n / $\langle d \rangle_n \geq 0.5$) and their shapes quite different from that of a LogNormal distribution, which is characterized by long decaying tails toward large sizes. Conversely, the Weibull distribution exhibits long tails toward small sizes and the Exponential distribution exhibits no tails at all at small sizes. For these two examples, the DEBUSSY method wildly fails in recovering the number distributions with errors on $\langle d \rangle_n$ and σ_n of $\sim 20\%$, but recovers the mass distribution with a somewhat satisfactory accuracy so that, in spite of the fact the PDS shape does not match accurately the expected one, all the mass parameters $\langle d \rangle_m$ and σ_m are retrieved with rather high accuracy (a few percents) except for σ_m of the Weibull distribution ($\sim 8\%$). Therefore, the DEBUSSY analysis appears to be reliable when dealing with mass distributions, but any comparison with other techniques based on the analysis of number PSDs (such as TEM or other optical microscopy methods) must be taken with care.

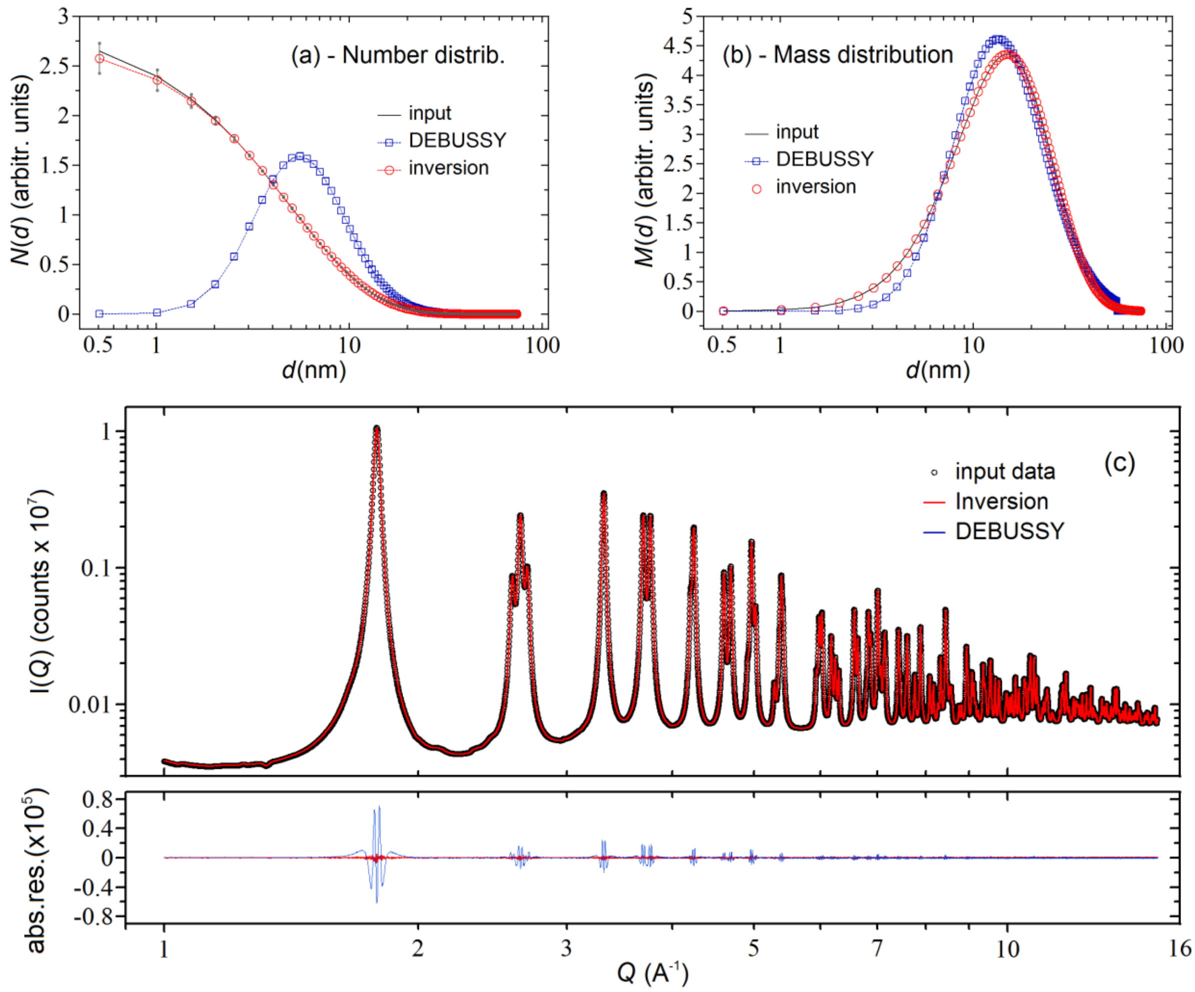

Figure S10 – (a) Simulated Exponential *number* input distribution of anatase TiO_2 nanocrystals with nominal $\langle d \rangle_n = \sigma_n = 5\ nm$ and corresponding (averaged) recovered distributions obtained with the DEBUSSY analysis (blue squares) and with our inversion algorithm (red circles). Statistics was accumulated by processing 100 noisy data signals with $SNR \sim 300.$; (b) corresponding input and recovered *mass* distributions; (c) Simulated input WAXTS (black circles) and reconstructed data obtained with the DEBUSSY analysis (blue line, not visible)and with our inversion algorithm (red line); (d) absolute residuals (recovered-input) for the data of panel c. DEBUSSY residuals are systematic and much higher than inversion residuals.

TABLE S5: comparison between input parameters of the Exponential *number* distribution of Fig.10 and the parameters recovered by using our inversion method and the DEBUSSY analysis. Three different SNR levels are shown.

	$<d>_n$ (nm)	σ_n (nm)	$<d>_m$ (nm)	σ_m (nm)	GOF
Input	5.258	4.998	19.986	9.957	
Inversion (SNR = 300)	5.30 ± 0.06	5.02 ± 0.01	19.998 ± 0.002	9.96 ± 0.01	1.00 ± 0.01
DEBUSSY (SNR = 300)	8.627 ± 0.004	5.060 ± 0.001	20.169 ± 0.001	10.270 ± 0.002	3.15 ± 0.01
Inversion (SNR = 100)	5.29 ± 0.17	5.00 ± 0.03	19.99 ± 0.01	9.97 ± 0.02	1.00 ± 0.01
DEBUSSY (SNR = 100)	8.63 ± 0.01	5.061 ± 0.002	20.17 ± 0.02	10.27 ± 0.02	1.40 ± 0.01
Inversion (SNR = 30)	5.32 ± 0.29	5.00 ± 0.05	19.99 ± 0.01	9.99 ± 0.06	1.00 ± 0.01
DEBUSSY (SNR = 30)	8.63 ± 0.02	5.064 ± 0.005	20.20 ± 0.03	10.30 ± 0.04	1.04 ± 0.01

Clearly, had we used in the DEBUSSY analysis the correct shape for the PSD, we would had found much more accurate results, consistent with the ones obtained with the inversion algorithm. It should be also pointed out that, regardless of the shape, when the PSDs are rather narrow ($\sigma_n / \langle d \rangle_n \leq 0.1$) the differences between our inversion method and the DEBUSSY analysis become more and more negligible. Under these circumstances, the DESUSSY analysis in more convenient because much faster than the inversion algorithm.

7) Synchrotron WAXTS data collection and reduction.

Magnetite-Maghemite (MM, Fe_3O_4 - γ-Fe_2O_3) and Titania (TiO_2) powder samples were loaded into borosilicate glass capillaries with certified composition (Hilgenberg GmbH 0500), 0.3mm and 0.5mm in diameter, respectively.

High-resolution Wide Angle X-ray Total Scattering (WAXTS) measurements were performed at the MS-X04SA Powder Diffraction Beamline of the Swiss Light Source (Paul Scherrer Institute, Villigen, CH).[1] Two different beam energies of 15 KeV (Fe_2O_3) and 17 KeV (TiO_2) were set and the operational wavelengths (λ_{15KeV} = 0.82712 Å, λ_{17KeV} = 0.70880 Å) accurately determined using a silicon powder standard (NIST 640d, a_0 = 0.543123(8) nm at 22.5°C). Data were collected in the 0.5°-130° 2θ range using a single-photon counting silicon microstrip detector (MYTHEN II).[2]

The spatial coherence length of the X-ray beam of the MS-X04SA beamline is claimed to be, in the longitudinal direction, of the order of 10^5 λ's, i.e., a few microns, and, in the transversal plane, up to 0.1 mm. Such coherence is much larger than the sizes of nanoparticles (< 100 nm) treated with DSE equation and the inversion algorithm (Eq.s 1 and 2 of the main text). Thus, the impinging field does not suffer of significant spatial variations and does not affect the analysis.

He/air background and empty glass capillaries were independently collected under the same experimental conditions. Additionally, an amorphous ferrihydrite sample was measured in the same experimental conditions used for the MM nanocrystals (NCs), to be added as background curve during the modelling.

Angle-dependent intensity corrections[3] were applied to the raw data to account for signal attenuations due to absorption effects; sample absorption curves were determined by measuring the transmitted beam from the filled capillaries, while for the empty capillaries the X-ray attenuation coefficient was computed using their nominal composition. Angular calibrations were applied to the zero angle and to x, y capillary offsets, derived from the certified silicon powder standard (NIST 640d) using locally developed procedures. Air and (absorption-corrected) capillary scattering contributions were subtracted from the signals of the samples.

8) Modeling and scattering profiles of Magnetite-maghemite (Fe_3O_4 - γ-Fe_2O_3) nanocrystals

The kernels profiles $I_p(Q_i, d_{p,j})$ used for the inversion algorithm were computed using the DEBUSSY Suite, a suite of programs implementing the Debye Scattering Equation (DSE) to model total scattering data of nanosized and disordered materials.[4] The Suite relies on a bottom-up approach that consists of two main steps. In the first one, a monovariate population of atomistic models of nanocrystals (NCs) with

increasing size and desired shape are generated, with the set of multiplicities of sampled interatomic distances stored in suitable databases. In the second step, this stored information is used to compute, via the DSE equation, the kernels $I_p(Q_i, d_{p,j})$.

Magnetite and maghemite are characterized by the same spinel-like crystal structure (space group Fd-3m), shown in Fig.S11, with oxygens forming a face centered cubic lattice and two crystallographic independent Fe atoms occupying octahedral and tetrahedral interstitial sites, respectively. In magnetite (Fe_3O_4, containing Fe^{2+} and Fe^{3+} in the 1:2 ratio) half Fe^{3+} ions show a tetrahedral coordination, whereas the other half, together with Fe^{2+} cations, occupy the octahedral sites. When magnetite is partially oxidized to maghemite (γ-Fe_2O_3), some additional iron vacancies are created. The crystal structure remains the same with a slight shrinking of the lattice ($a = 8.397$ Å for magnetite to $a = 8.346$ Å for maghemite).[5]

The DSE modelling strategy behind the calculation of the Magnetite-Maghemite (MM) kernel functions was similar to the one used in Ref.[5], but based on the use of constant (not size dependent) crystallographic parameters. In particular:

(i) the cubic crystal structure was built with an average lattice parameter $a = 8.36052$ Å, which is in between the ones characterizing the magnetite and maghemite structures. This figure (slightly expanded with respect to that of magnetite[5]) accounts for surface expansion effects that are common in many oxides and are mainly due to repulsion between unsaturated ions in the NCs shell.[6] Based on this

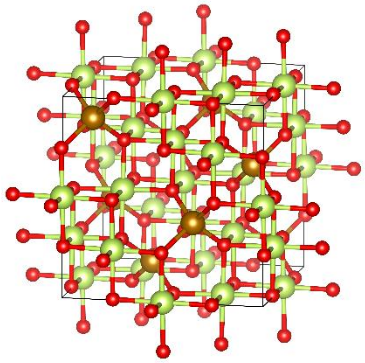

Figure S11. - Crystal structure of Fe_3O_4 and γ-Fe_2O_3. Oxygen ions are in red, iron ions in tetrahedral sites [$Fe_{(tet)}$] in gold and iron ions in octahedral coordination [$Fe_{(oct)}$] in light green.

structure, a monovariate population of NCs clusters of spherical shape and increasing diameter ($\Delta d = 0.65 nm$) were generated up to $d = 50\ nm$. The corresponding multiplicities of the sampled interatomic distances of each cluster were stored a suitable database.

(ii) DSE calculations were carried out by using constant site occupancy and Debye-Waller factors. The site occupancy factors (*s.o.f.*) for the iron atom in the octahedral sites was set to s.o.f.(Fe_{oct}) = 0.89, so to account for iron vacancies originating from the maghemite formation, likely at the NCs surface; all the other site occupancy factors were kept at 1.0. Debye-Waller factors of 0.46 $Å^2$, 0.91 $Å^2$ and 0.33 $Å^2$ were used for $Fe_{(tet)}$, $Fe_{(oct)}$ and O, respectively. These values have been derived as average parameters from the size dependent function described in Ref.[5]

9) Modeling and scattering profiles of commercial Titania (TiO_2) nanocrystals.

Two spherical databases for the two polymorphs of TiO_2 (anatase and rutile) were built, implementing the same strategy detailed for MM NCs and using the structural parameters available in literature,[7] further optimized by a Rietveld refinement using the Topas program:[8] *a* = 3.78596 Å and *c* = 9.50805 Å for anatase (space group $I4_1$/amd); *a* =4.59469 Å and *c* =2.95921 Å for rutile (space group $P4_2$/mnm).

The TiO_2 kernel profiles $I_p(Q_i, d_{p,j})$ for both phases were computed by using the DEBUSSY suite with the exact DSE (Eq.1) for spherical NCs up to diameters of $d{\sim}80\ nm$. Above this size, the computation via the DEBUSSY Suite become rather impractical because of very long computational times.

While for the anatase phase sizes up to $80\ nm$ are large enough to recover correctly the PSD, the recovered distribution of the rutile appears to be truncated and suggests the presence of larger sizes. Thus we resorted to an alternative approach based on (Rietveld-inspired) analytical pseudo-Voigt functions describing the shapes of the diffraction peaks and a polynomial description of the diffuse scattering hidden in the background baseline. All these parameters were derived upon calibration using the DSE signals for the smaller NCs (up to ca. $d = 80nm$) as benchmarks (shown in Fig.S12).

Once derived through calibration, these parameters were used to calculate, using the TOPAS program,[8] all the kernels for rutile used in the inversion algorithm, up to $d \sim 200nm$.

The same Debye-Waller factor (0.6Å^2) and *s.o.f.*=1.0 were used for both atomic species (Ti and O) and both phases (anatase and rutile). For both phases, the inelastic Compton scattering contribution has been added as an additional model component at the final simulated patterns.

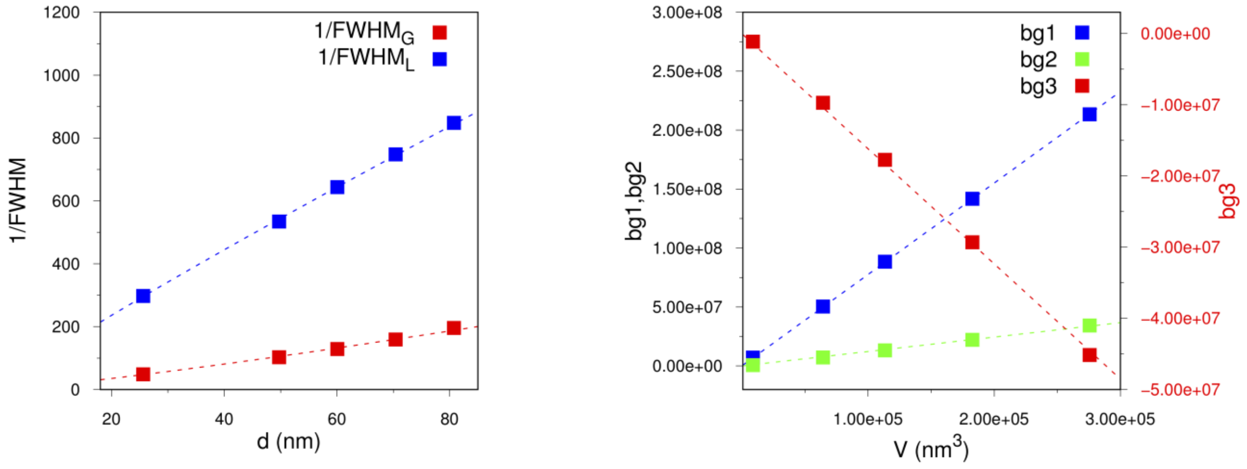

Figure S12 - (a) Gaussian (1/FWHM$_G$) and Lorentian (1/FWHM$_L$) "apparent" crystal sizes as a function of the clusters diameter d, derived using pseudo-Voigt functions describing the peaks width of the DSE reference patterns, for each cluster selected for the calibration (up to *d* = 80nm). The dotted lines are the fitting curves [$1/FWHM_G(d)=0.969d^{1.2001}$ and $1/FWHM_L(d)= 15.321d^{0.9129}$)] used for extrapolating the FWHM$_G$ and FWHM$_L$ values at *d* > 80nm. (b) Three background parameters (bg1, bg2, bg3), used for describing the diffuse scattering contribution in the Rietveld-like fits for *d* < 80nm and extrapolated at higher values, as a function of the clusters volume V. The dotted lines are the extrapolating curves [b1(V)=775.6V; b2(V)=122.49V; b3(V)=-161.75V].

10) Inversion from multimodal distributions.

In this section we show two examples of the capability of our method in recovering bi-modal distributions that were characterized by two peaks of different widths and relative heights. Figure S13 reports the case of a bi-Gaussian distribution of anatase TiO_2 nanocrystals characterized by two broad peaks, namely $< d_1 >_m = 6.88nm$, $\sigma_{1m} = 1.75nm$, mass fraction $w_1 = 0.15$ and by $< d_2 >_m = 23.4nm$, $\sigma_{2m} = 4.65nm$ and mass fraction $w_2 = 0.85$. The noisy data ($SNR = 300$) were inverted by setting $\omega_p = 2 \times 10^{-4}$ and the iterative procedure was stopped after $\sim 7.2 \times 10^4$ iterations when the relative variation of GOF attained the threshold $\delta(r) \leq 10^{-9}$(see step (d) of section 4) and GOF~ 1.01. As one can notice, the matching between the input (black solid curves) and the recovered (symbols) distributions is excellent, for both number (Fig.S13a) and mass (Fig.S13b) distributions.

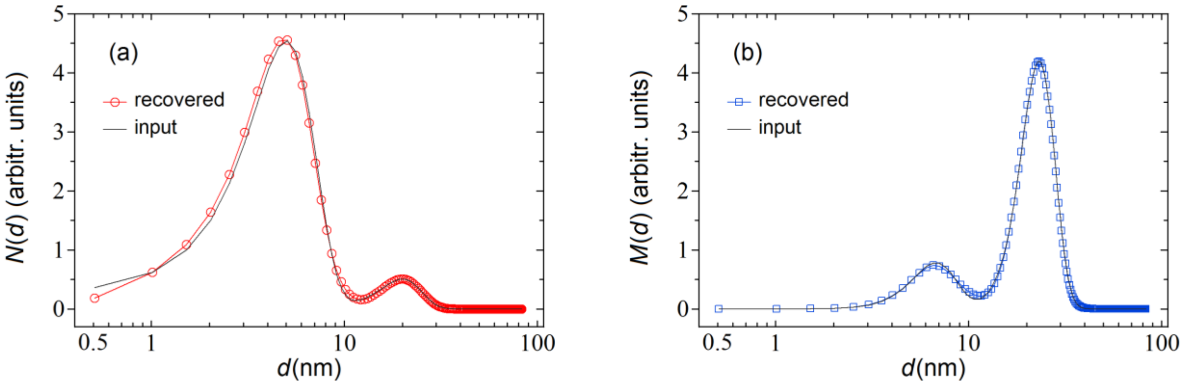

Figure S13 – (a) Simulated Bigaussian *number* input (solid line) distribution of anatase TiO_2 nanocrystals characterized by two broad peaks (see text) and corresponding recovered distribution obtained with our inversion algorithm (red circles); (b) corresponding input (solid line) and recovered *mass* distributions (blue squares).

Figure S14 reports the case of a bi-Gaussian distribution of anatase TiO_2 nanocrystals characterized by narrow peaks, namely $< d_1 >_m = 20.0nm$, $\sigma_{1m} = 1.0nm$, mass fraction $w_1 = 0.54$ and by $< d_2 >_m = 30.0.6nm$, $\sigma_{2m} = 1.0nm$ and mass fraction $w_2 = 0.46$. The noisy data ($SNR = 300$) were inverted by setting $\omega_p = 1 \times 10^{-6}$ and the iterative procedure was stopped at the maximum number of iterations 10^6 where GOF$\sim$0.99. As for the case of Fig.S13, the matching between the input (black solid curves) and the recovered (symbols) distributions is excellent, for both number (Fig.S14a) and mass (Fig.S14b) distributions.

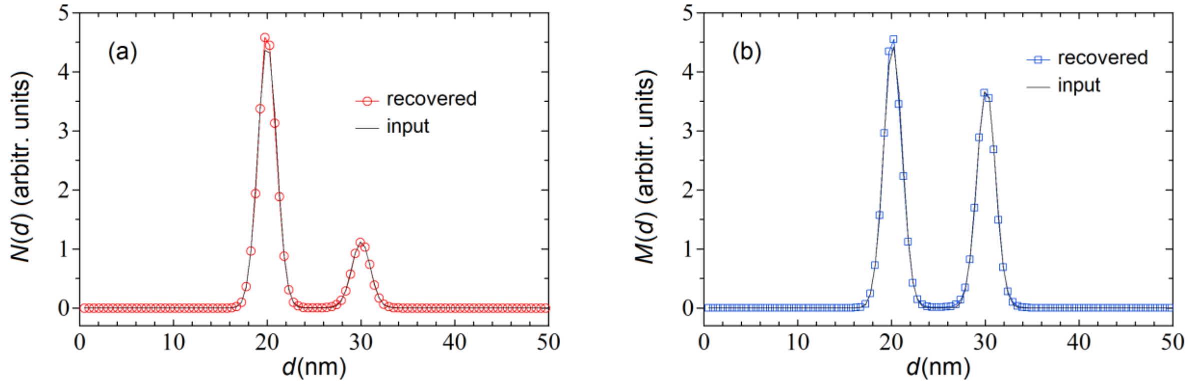

Figure S14 – (a) Simulated Bigaussian *number* input (solid line) distribution of anatase TiO_2 nanocrystals characterized by two narrow peaks (see text) and corresponding recovered distribution obtained with our inversion algorithm (red circles); (b) corresponding input (solid line) and recovered *mass* distributions (blue squares).

11) Ill-posedness analysis of the WAXTS-DSE data inversion problem.

We show in this section that the inversion of WAXTS-DSE data is not a severely ill-posed problem. Indeed, as mentioned in the main text, the kernels $I_p\left(Q_i, d_{p,j}\right)$ associated to Eq.(1) are highly structured with the presence of a large number of relatively narrow and differently shaped peaks, whose amplitude, width, and positions depend sensitively on NC size and morphology. Thus, as long as the data $I_m(Q_i)$ are taken over a large Q-range with a high Q-resolution and high SNR ratio, the difference of the Intensity profiles of two adjacent size classes is higher than noise and the inversion algorithm can recover the correct distribution without introducing artefacts. An example of the dependence of the intensity profiles for 7 anatase TiO2 NCs with sizes ranging between 0.5 and 83 nm, is reported in Fig.S15. As one can appreciate, the average intensity, peaks height and widths vary sensitively with the NCs sizes.

A quantitative analysis of this behaviors is reported in Fig.S16, where we show that the average intensity scales as $\langle I(Q)\rangle \sim d^3$ [Fig.S16(a)], the peak intensity as $I_{peak} \sim d^4$, [Fig.S16(b)], the peak width as $I_{FWHM} \sim d^{-1}$, [Fig.S16(c)] and the diffuse scattering around the peaks as $I_{diff} \sim d^3$, [Fig.S16(d)]. All this behaviors are characterized by a high dynamic range of variation, implying that kernels of Eq.(1) is highly sensitive to the NC sizes.

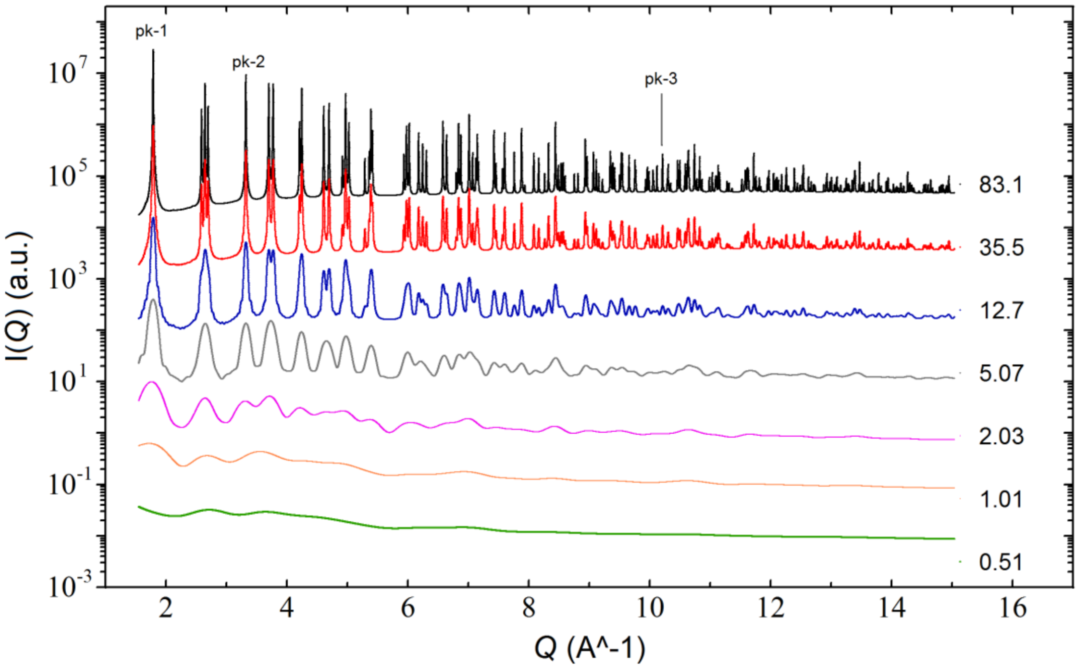

Figure S15 – Behaviors of the kernels $I_p(Q_i, d_{p,j})$ associated to Eq.(1) of the main text for 7 anatase TiO_2 NCs with diameters ranging between 0.51 and 83.1 nm. Passing from the smallest to the largest sizes, the intensity profiles vary by many orders of magnitude and the peaks become increasingly high and narrow.

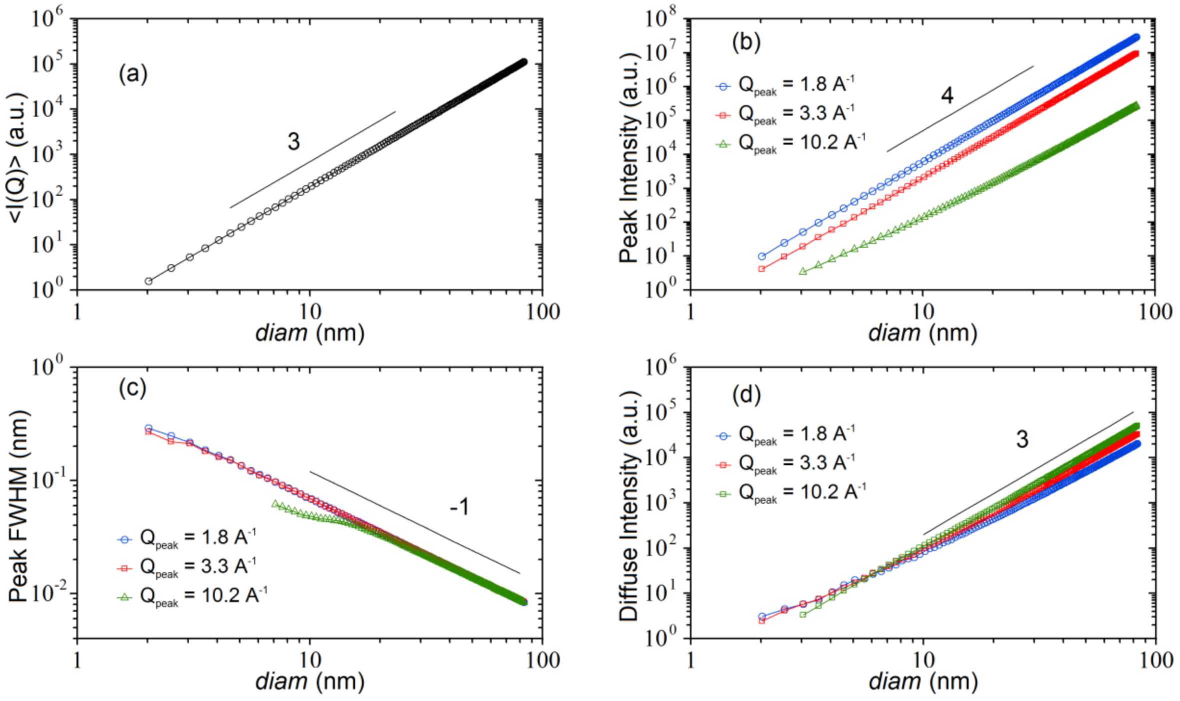

Figure S16 – Behaviors, as a function of the diameter, of the features characterizing the anatase TiO_2 NCs kernels of Fig.S15. (a) average intensity; (b) peak intensities of three peaks indicated by the labels of Fig.S15; (c) peak FWHMs; (d) intensity of the diffuse scattering around the peaks.

As already mentioned above, the ill-posedness of an inversion problem is highly related to the noise level present on the data in comparison with the (*r.m.s.*) difference between the kernels of two adjacent sizes. Fig.S17 shows the relative difference between three couples of adjacent kernels spanning the entire range of the anatase TiO_2 sizes used in the reconstruction. Before taking the difference, the intensity profile of each kernel has been normalized to $(d_j)^3$, so that all the kernels are rescaled to the same average intensity. As one can notice, the relative difference is pretty large for the smaller sizes (kernels 4 and 5 with $d_4 = 2.02nm$ and $d_5 = 2.53nm$), whereas becomes increasingly smaller for large sizes (kernels 163 and 164 with $d_{163} = 82.60nm$ and $d_5 = 83.11nm$). The equivalent SNR_{eqv} associated to such a difference is $SNR_{eqv} = \left(\sum_{i=1}^{N}\langle I\rangle_i^2 / \sum_{i=1}^{N}\Delta I_i^2\right)^{1/2}$ where $\langle I\rangle_i = [I_1(Q_i) + I_2(Q_i)]/2$ and $\Delta I_i = I_2(Q_i) - I_1(Q_i)$ (the suffixes 1 and 2 indicating the members of the couple). Such figures, indicated in the legend of Fig.S17, are smaller than the SNR of the data for each couple of adjacent kernels spanning the entire size range. Thus, the inversion problem is expected to be not severely ill-posed.

Finally, when the kernels of different polymorphs are compared, the peaks of the intensity profiles show up at rather different Q values (occasionally overlapping, particularly at high Q's), implying a very high (up to $\sim 100\%$) relative differences. Correspondingly, the SNR_{eqv} values of couple of kernels of distinct polymorphs are very low, and the problem is clearly not ill-posed.

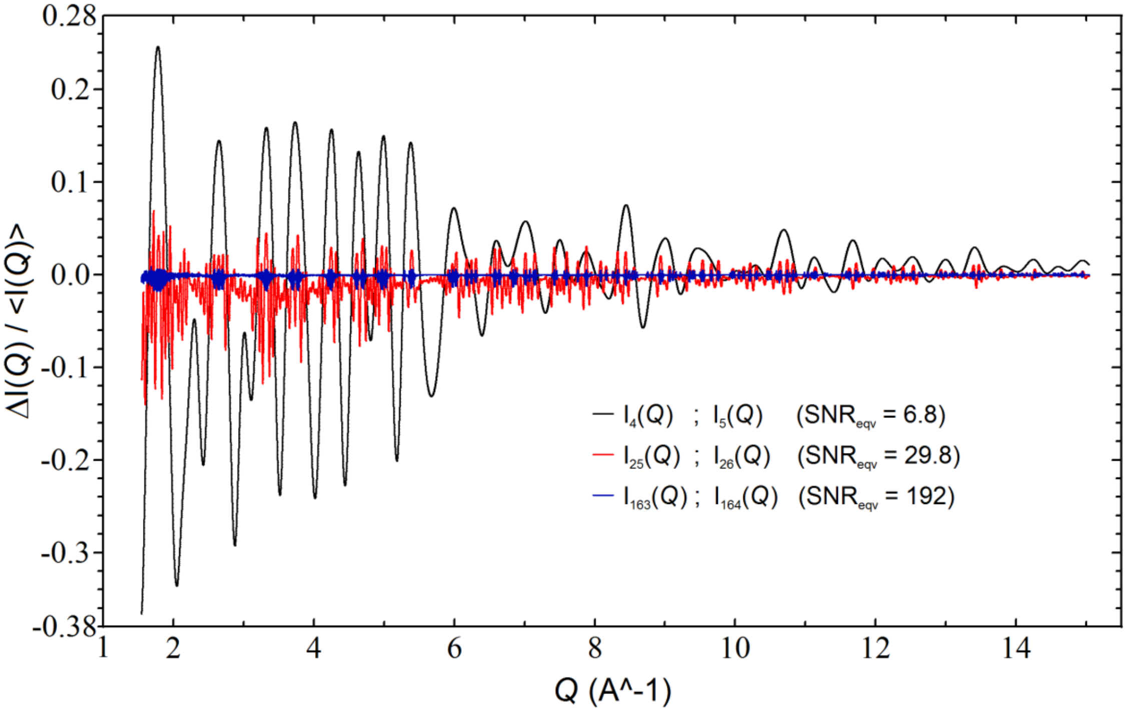

Figure S17 – relative difference between three couples of adjacent kernels for the anatase TiO_2 NCs of Fig.S15. Small sizes exhibit relative differences much higher than those associated to large sizes. Their corresponding SNR_{eqv} (see text) are always smaller than typical SNR present on the data.

References

[1] P.R. Willmott et al., *J. Synchrotron Radiat.* 2013, **20**, 667-682

[2] A. Bergamaschi et al., *J. Synchrotron Radiat.* 2010, **17**, 653-668.

[3] M. Bowden and M. Ryan, *J. Appl. Cryst.,* 2010, **43**, 693-698.

[4] A. Cervellino, R. Frison, F. Bertolotti and A. Guagliardi, *J. Appl. Cryst*. 2015, **48**, 2026-2032.

[5] R. Frison et al., *Chem. Mater*. 2013, **25**, 4820-4827.

[6] P. M. Diehm, P. Ágoston, K. Albe, Chem. *Phys. Chem*., 2012, **13**, 2443–2454.

[7] C.J. Howard, T.M. Sabine and F. Dickson, *Acta Cryst*. *B*., 1991, **46**, 462-468.

[8] TOPAS v3.0, 2005, Bruker AXS, Karlsruhe, Germany.
